# Antibiotic susceptibility signatures identify potential antimicrobial targets in the *Acinetobacter baumannii* cell envelope

**DOI:** 10.1038/s41467-020-18301-2

**Published:** 2020-09-09

**Authors:** Edward Geisinger, Nadav J. Mortman, Yunfei Dai, Murat Cokol, Sapna Syal, Andrew Farinha, Delaney G. Fisher, Amy Y. Tang, David W. Lazinski, Stephen Wood, Jon Anthony, Tim van Opijnen, Ralph R. Isberg

**Affiliations:** 1 Department of Biology, Northeastern University, Boston, MA 02115 USA; 2 Department of Molecular Biology and Microbiology, Tufts University School of Medicine, Boston, MA 02111 USA; 3 Laboratory of Systems Pharmacology, Harvard Medical School, Boston, MA 02115 USA; 4 Biology Department, Boston College, Chestnut Hill, MA 02467 USA

**Keywords:** DNA replication, Antimicrobials, Cellular microbiology, Microbial genetics

## Abstract

A unique, protective cell envelope contributes to the broad drug resistance of the nosocomial pathogen *Acinetobacter baumannii*. Here we use transposon insertion sequencing to identify *A. baumannii* mutants displaying altered susceptibility to a panel of diverse antibiotics. By examining mutants with antibiotic susceptibility profiles that parallel mutations in characterized genes, we infer the function of multiple uncharacterized envelope proteins, some of which have roles in cell division or cell elongation. Remarkably, mutations affecting a predicted cell wall hydrolase lead to alterations in lipooligosaccharide synthesis. In addition, the analysis of altered susceptibility signatures and antibiotic-induced morphology patterns allows us to predict drug synergies; for example, certain beta-lactams appear to work cooperatively due to their preferential targeting of specific cell wall assembly machineries. Our results indicate that the pathogen may be effectively inhibited by the combined targeting of multiple pathways critical for envelope growth.

## Abstract (web summary)

A unique cell envelope contributes to the antibiotic resistance of the pathogen Acinetobacter baumannii. Here, Geisinger et al. identify A. baumannii mutants with altered antibiotic susceptibility, infer the function of uncharacterized proteins involved in envelope synthesis, and predict antibiotic synergies.

## Introduction

The World Health Organization, Food and Drug Administration, and Centers for Disease Control each rank restriction of *Acinetobacter baumannii* as among the most critical targets for developing new antimicrobials^1–3^. This Gram-negative rod causes drug-resistant nosocomial diseases in the critically ill, commonly manifesting as bloodstream infections and ventillator-associated pneumonia^4^. Resistance to an extensive range of antibiotics, including formerly last-resort agents such as carbapenems, is now widespread among *A. baumannii* isolates, with the emergence of strains resistant to all available antibiotics now documented^5,6^. Few therapeutic options remain to control this threat.

A better understanding of what makes *A. baumannii* so difficult to treat is critical for improved strategies that attack the pathogen. The evolution of drug resistance in *A. baumannii* in large part is due to acquisition of inactivating enzymes or drug target mutations blocking antibiotic lethal action^7,8^. These acquired alterations, which vary across isolates, act in concert with conserved mechanisms tightly linked to reduced drug penetration, including a low-permeability cell envelope and upregulation of efflux pumps^9,10^. Insight into the intrinsic envelope-level defenses has the potential to inform ways to enhance antibiotic killing across diverse isolates.

A powerful approach to revealing the genetic contributions to intrinsic mechanisms of drug defense is via high-density knockout mutant libraries, which allow measurement of genotype–phenotype relationships on a genome-wide scale^11,12^. This approach has been used to identify genes modulating susceptibility to a variety of antibiotic stresses^13–18^, and has been used to identify intrinsic defenses against a selection of antimicrobial treatments in *A. baumannii*^9,19,20^. Despite the utility of these approaches in measuring gene–antibiotic interactions, understanding the mechanisms behind the uncovered resistance determinants is limited by difficulties associated with providing accurate gene annotations. A large fraction of genes in any organism lack characterization and have no known or predicted function (referred to as “orphan” or “hypothetical” genes)^13,21,22^. Lack of functional information complicates downstream analyses, and single gene–antibiotic phenotypes can be insufficient to generate hypotheses on function. Moreover, in species divergent from model organisms, functional annotations predicted by sequence homologies are often inaccurate, as the function of sequence orthologs may not be conserved^22,23^. Hypothetical genes lacking annotation and genes with inaccurate annotation due to noncanonical functions are predicted to be particularly problematic with *Acinetobacter*, which has diverged from other γ-proteobacteria and lacks many canonical proteins that function in envelope biogenesis^10,24^. A notable area of divergence involves processes related to cell division. Key proteins that coordinate cell envelope ingrowth and septum formation with chromosome replication (such as ZapB and SlmA) and cell separation (FtsEX) have no orthologs in *Acinetobacter*^24^.

In this paper, we comprehensively characterize mechanisms of intrinsic defense in *A. baumannii* against multiple antibiotics via transposon sequencing (Tn-seq) and leverage the diversity of phenotypes generated to address the problem of uncharacterized gene function in this pathogen. By analyzing the patterns of altered antibiotic susceptibility caused by gene-inactivating mutations across the genome, we uncover functional relationships of conserved hypothetical proteins with key cell biological processes and expand the roles of annotated enzymes in envelope synthesis. The identified determinants of susceptibility represent novel targets for potentiating current antibiotics against *A. baumannii*. Moreover, this analysis informs a strategy to combine different β-lactam antibiotics for enhanced antimicrobial activity.

## Results

### Defining drug susceptibility determinants in *A. baumannii*

**Fig. 1:**
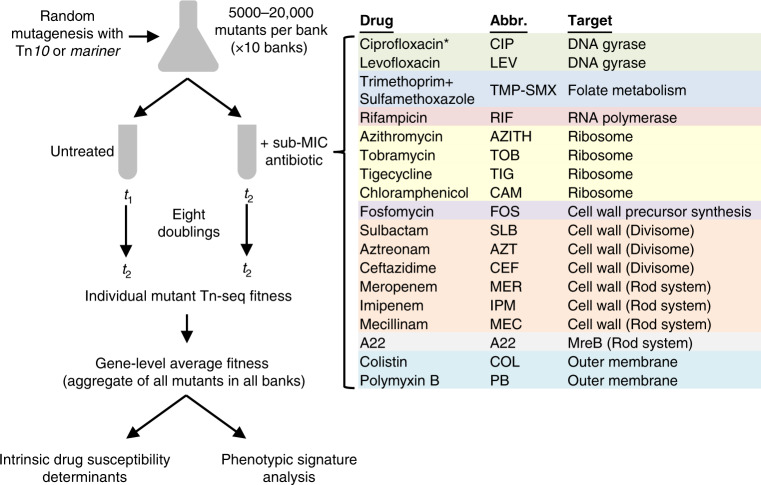
Genome-wide profiling of gene–antibiotic interactions in *A. baumannii* by Tn-seq. Diagram outlines the multiple parallel Tn-seq fitness profiling experiments as described in “Methods”. Sub-MIC drug concentrations used to achieve 20–30% growth rate inhibition are listed in Supplementary Table 1. For each antibiotic, genes contributing to intrinsic defense against each single drug were identified by using significance criteria (Methods). Fitness profiles across all conditions (phenotypic signatures) were then analyzed to identify novel gene relationships and discover multicondition discriminating genes. Drug abbreviations (Abbr.) and targets are listed. *CIP Tn-seq data used in these studies were described previously^20^.

To examine the genome-wide molecular mechanisms that modulate antibiotic action in *A. baumannii*, we measured the effects of transposon insertion mutations on bacterial growth during challenge with a broad set of antimicrobial compounds by Tn-seq^12^. Antibiotics were selected that target a variety of essential cellular processes, with about half of the treatments targeting the cell envelope (Fig. 1). This subset includes antibiotics that target distinct aspects of cell wall biogenesis governing elongation or division. In addition to defining elements of intrinsic drug susceptibility, the use of multiple distinct stress conditions facilitates the determination of specific fitness phenotypes for a large swath of genes in *A. baumannii*. The relatedness of these fitness profiles is predicted to provide leads regarding the function of uncharacterized proteins that contribute to drug resistance.

To measure the effect of each antibiotic on relative fitness of transposon mutants, we used previously constructed random banks of *A. baumannii* ATCC 17978 Tn*10* insertion mutants^20^ as well as random banks of *Himar1 mariner* mutants constructed for these purposes (Methods). For each transposon, 10 independent insertion pools, each consisting of 5000–20,000 unique mutants (>60,000 mutants in total with Tn*10*, >85,000 mutants with *mariner*), were seeded in rich broth at *A*_600_ 0.003 (approximately, 2.5 × 10^6^ CFU/ml) in the presence or absence of antibiotic. Antibiotics were used  below the minimal inhibitory concentrations (MIC) such that growth rate was lowered by 20–30% compared to growth without antibiotics (Supplementary Table 1), a degree of selective pressure enabling detection of mutants with altered susceptibilities^20^. In the case of sulbactam (SLB; all abbreviations listed in Fig. 1 and Supplementary Table 1), an important component of empiric antibiotic therapy for *A. baumannii* infections, we tested an additional, lower concentration resulting in 10–15% growth inhibition that should detect only the strongest elements of intrinsic resistance to the drug. Both treated and untreated cultures were allowed to grow to *A*_600_ 0.5–1 (8 doublings). DNA was isolated from samples taken at the start and end of this outgrowth, and transposon insertion sites were polymerase chain reaction (PCR)-amplified and enumerated by massively parallel sequencing. Read counts mapping to the chromosome and plasmid pAB3 were used to calculate a normalized value of the fitness of each individual transposon mutant relative to the entire pool using established methods^20^. Fitness data across all pools from all mutants mapping to the same gene were then aggregated to generate a gene-level fitness value (Supplementary Data 1), which was used to assess the contribution of each gene to antibiotic-specific growth.

The population-wide Tn-seq fitness method incorporates information from two points in growth, so the effects of chromosome position bias observed previously^25^ are largely negated. An exception was the aminoglycoside tobramycin (TOB), which caused chromosome origin-proximal genes to show higher average fitness scores than those of terminus-proximal genes (Supplementary Fig. 1). To eliminate position bias, fitness values from TOB treatment were normalized by fitting to a locally weighted scatterplot smoothing (LOWESS) curve. The other case of position bias was seen with the fluoroquinolone levofloxacin (LEV), but fitness values were associated with the region of two prophages (Supplementary Fig. 1, red arrowheads). We demonstrated previously that these increases are associated with a DNA gyrase block in a fluoroquinolone-sensitive background, and the LEV data here mimic the position-specific fitness data observed previously with ciprofloxacin (CIP)^20^.

From the gene-level Tn-seq fitness data, we identified candidate determinants of susceptibility to each antibiotic (Supplementary Data 2). These determinants were defined using previously described criteria^20^: (1) gene-level Tn-seq fitness was at least 10% lower or higher with antibiotic challenge compared to untreated control, (2) fitness difference had a *q* value (false-discovery rate) <0.05, and (3) fitness was calculated from *n* ≥ 3 independent mutants. When considering all 20 antibiotic stress conditions, including previously described data with 2 doses of CIP^20^, we identified 327 candidates determining susceptibility to at least 1 antibiotic treatment (Supplementary Fig. 2, blue data points; Supplementary Data 2). Ten of these genes determined susceptibility to at least half of the 20 treatments (Supplementary Table 2), indicating that these genes are associated with the ability of *A. baumannii* to cope with a broad range of stresses. Among the ten genes are known determinants of multidrug defense including each component of the *adeIJK* multidrug efflux system^26^, and the *bfmR* envelope regulator that we have previously shown modulates survival after antibiotic exposure^27^. Additional candidate broad susceptibility determinants controlling envelope-level processes were genes encoding the putative periplasmic protease CtpA^20,27^, lipooligosaccharide (LOS) synthesis enzymes LpsB and LpxL^28,29^, and BlhA, a protein of unknown function involved in cell division^20,30^. An uncharacterized gene present in plasmid pAB3 (ACX60_RS18565) was also detected as modulating defense against several antibiotics.

We examined previous transcriptomic data to determine whether BfmR deficiency was associated with decreased expression of any of the candidates that conferred broad susceptibility. Loss of BfmR caused a significant, twofold decrease in the expression of one of these determinants, *blhA* (Supplementary Fig. 2). Altered BlhA levels may be a contributor to BfmR-mediated defense against antibiotics but cannot explain the antibiotic hypersensitivity profile of *bfmR* mutations. Given the large BfmR modulon^27^ and the overlapping but distinct pattern of drug susceptibility observed with *bfmR* and *blhA* mutations (see Supplementary Table 2 and below), it is likely that BfmR-mediated resistance comprises the concerted regulation of multiple additional determinants.

While transposon mutation of most (245) of the 327 candidate susceptibility determinants caused reduced fitness with at least one drug, a number of these genes (122) showed increased fitness with at least one drug when mutated (Supplementary Data 2). These included known resistance modulators such as *adeN*, a negative regulator of *adeIJK*^26,31^, and *bfmS*, encoding the predicted cognate receptor controlling BfmR activity^27,32^. Mutations in nonessential genes encoding proteins involved in translation (including ribosome biogenesis, tRNA modification, and amino acid biosynthesis), 1,3-diaminopropane synthesis (*dat* and *ddc*)^33,34^, and cAMP degradation (*cdpA*)^35^ were also associated with increased fitness with antibiotic challenge. Many of the mutants showing enhanced fitness with antibiotic challenge had relatively low fitness in drug-free medium (Supplementary Fig. 2), consistent with an association between enhanced defense against antibiotic stresses and mutations causing reduced growth rate^20,36,37^.

### Drug susceptibility signatures reflect gene connectivity

We predicted that altered susceptibilities to antibiotics could be used to identify functional relationships among *A. baumannii* genes. To this end, we generated a phenotypic signature for each gene^13^ by compiling the gene-level fitness values from all tested conditions, including the 20 antibiotic stresses and 12 untreated controls (Supplementary Data 1). To maximize analysis of variation across conditions, fitness values were scaled such that they represented the change from mean fitness in standard deviation units (*z*-scores).

**Fig. 2:**
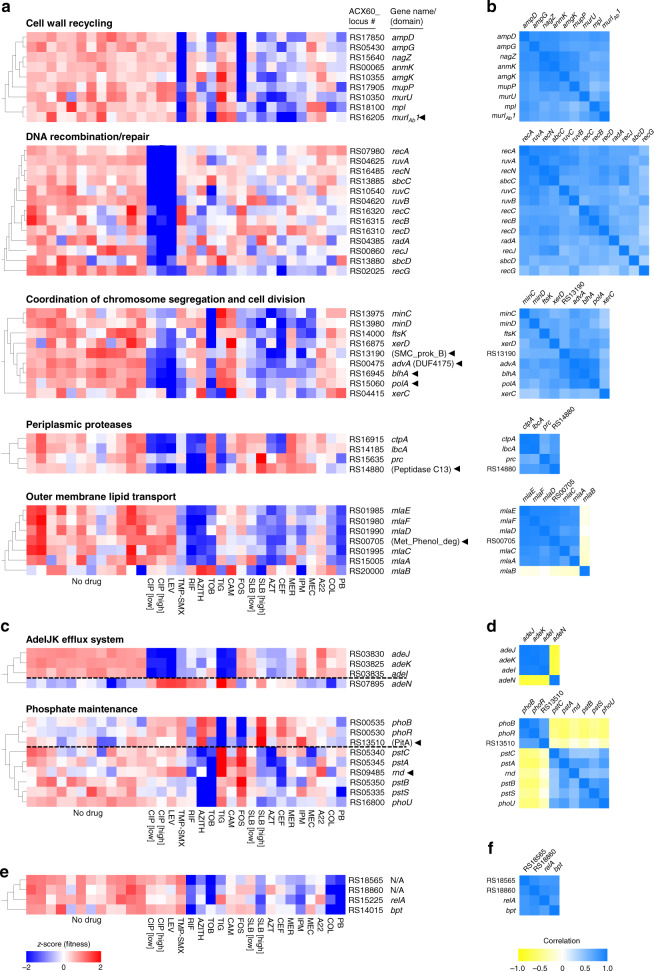
Genes with interconnected functions show correlated phenotypic signatures. **a**–**f** Genes within a shared functional pathway show relationships in their Tn-seq fitness signatures. **a**, **c** Heat maps (using blue-white-red scale at bottom) show normalized Tn-seq fitness in *z*-scored units for mutants in each gene (rows) grown in distinct conditions (columns). Characterized/annotated genes were first placed into shared functional pathways (Methods). We next identified additional genes (arrowheads), many of which were uncharacterized/unannotated, that correlate with each pathway by using hierarchical clustering with the entire set of *A. baumannii* phenotypic signatures. Parentheses denote domains identified from NCBI conserved domain database (CDD)^46^. In **c**, dashed black lines separate genes with opposing regulatory effects. **b**, **d** Heat maps (using yellow-white-blue scale at bottom) show Pearson correlation coefficient (*r*) matrices measuring relatedness of the corresponding Tn-seq fitness signatures in (**a**). Positive and negative *r* indicate correlation and anticorrelation, respectively. **e**, **f** Phenotypic signatures correlating with pAB3-encoded ACX60_RS18565. Tn-seq fitness (**e**) and correlations (**f**) are shown as above. N/A, no gene name or predicted protein domain. Source data are provided as a Source Data file.

The data analyzed in this fashion indicate that drug susceptibility phenotypic signatures can effectively identify gene relationships. First, sets of well-annotated genes whose products physically interact or perform functions within a shared pathway based on experimentation or inferred from orthology (Methods) show phenotypic signatures that are highly correlated (Fig. 2a–d, genes not marked by arrowheads). For example, Tn-seq fitness profiles were significantly correlated for genes encoding proteins that function within cell wall recycling^38,39^ (*r* = 0.45–0.95, *p* < 0.009), periplasmic proteolysis^40^ (*r* = 0.58–0.98, *p* < 0.0006), DNA recombination and repair^20^ (except for some pairings with *recG*; *r* = 0.46–0.93, *p* < 0.009), and the Mla outer membrane (OM) lipid transport system^41–43^ (*mlaA* and *mlaC*-*F*) (*r* = 0.69–0.92, *p* < 0.0001) (Fig. 2a, b). The one exception to the Mla complex was *mlaB* (*r* = −0.01 to −0.21, *p* > 0.23), but *Escherichia coli* mutations in this gene are weak compared to mutations in other *mla* genes^42^. Genes encoding proteins that coordinate chromosome segregation with cell division^44^ also had significantly correlated signatures (*r* = 0.35–0.73, *p* < 0.05) (Fig. 2a, b). These signatures resembled those associated with DNA recombination and repair but were distinguished by concerted hypersensitivity to agents that attack the cell wall as well as to those that damage DNA (Fig. 2a). Second, genes with opposing activities show anticorrelated phenotypic signatures. For example, the phenotypic signature of *adeN* is highly anticorrelated with the signatures of the *adeIJK* efflux system, consistent with the regulator negatively controlling this operon^26,31^ (*r* = −0.80 to −0.77, *p* < 10^−6^) (Fig. 2c, d). A similar pattern was seen with genes associated with phosphate homeostasis. In many bacteria, the two-component system PhoBR transcriptionally regulates phosphate-acquisition in response to signals from the phosphate-sensing PstSCAB–PhoU complex^45^. Mutations in one system result in opposite effects on gene expression in the other in *E. coli*^45^. The phenotypic signatures of the transposon mutations in *A. baumannii* significantly correlated within each system (*phoBR*, *r* = 0.91, *p* < 10^−11^; *pstSCAB*-*phoU*, *r* = 0.4–0.81, *p* < 0.024) while between the two systems they anticorrelated (*r* = −0.42 to −0.62, *p* < 0.017) (Fig. 2c, d). Therefore, we expect that antibiotic–gene phenotypic signatures reflect underlying physical or functional connectivity, allowing new leads on gene function in *A. baumannii*.

As an initial test of this hypothesis, we performed hierarchical clustering of genome-wide phenotypic signatures to identify additional genes that correlate with the pathways highlighted in Fig. 2. Analysis of these pathways allowed identification of several co-clustering genes that had poor or no functional annotations (Fig. 2a, c, genes marked with arrowheads). Interestingly, a number of such genes had signatures matching those of the division-chromosome coordination pathways. These included two hypothetical genes encoding a DUF4175 domain of unknown function (ACX60_RS00475) or a structural maintenance of chromosomes domain (SMC_prok_B; ACX60_RS13190)^46^, the poorly understood gene *blhA*, as well as *polA* encoding DNA polymerase I (*r* = 0.46–0.94, *p* < 0003; Fig. 2a, b). Similarly, phenotypic signatures connected *mlaA*,*C*,*D*,*E* and *F* with an uncharacterized gene (ACX60_RS00705) encoding a protein with a Phenol_MetA_deg domain, which is part of a family of OM channel domains implicated in hydrophobic molecule uptake^47,48^ (*r* = 0.69–0.92, *p* < 10^−4^; Fig. 2a, b), consistent with this being an uncharacterized and potentially essential member of the Mla complex.

Encouraged by these results, we applied the same analysis to the other pathways. A gene (ACX60_RS14880) encoding a peptidase domain predicted to be periplasmic^49^ clustered with genes encoding the *A. baumannii* orthologs of periplasmic proteases CtpA, Prc, and the CtpA binding partner LbcA^40^ (*r* = 0.51–0.80, *p* < 0.004; Fig. 2a, b). ACX60_RS13510, encoding a predicted PitA-family phosphate transporter, was highly correlated with *phoBR* (*r* = 0.70–0.71, *p* < 10^−5^), while a Ribonuclease D ortholog (*rnd*) was found to cluster with *pstSCAB*-*phoU* (*r* = 0.46–0.72, *p* < 0.02) (Fig. 2c, d). In addition, one of two *murI* paralogs in *A. baumannii*^50,51^ had a phenotypic signature connected to PG recycling (*murI*_*Ab*_*1*, *r* = 0.52–0.79, *p* < 0.003; Fig. 2a, b). Finally, hierarchical clustering identified mutations that match the phenotypic signature of the pAB3-encoded broad antibiotic susceptibility determinant ACX60_RS18565 (Fig. 2e). These included mutants mapping to the *relA* ppGpp synthetase and to an ortholog of the *bpt* leucine aminoacyl protein transferase involved in N-end-rule degradation^52,53^ (*r* = 0.72–0.87, *p* < 10^−5^, Fig. 2f). Together, these results illustrate the ability of antibiotic sensitivity changes to identify leads on the functions of poorly characterized genes based on phenotypic signatures.

### AdvA is a critical cell division protein in *A. baumannii*

The cluster analysis showing close relationships between ACX60_RS00475 and genes associated with chromosome replication/segregation and cell division (Fig. 2a) predicted a related function for this uncharacterized protein in *A. baumannii*. As the pathogen lacks orthologs of several canonical proteins controlling these pathways (FtsE and FtsX, and Z-ring modulators ZapB, SlmA, and SulA)^24^, we hypothesized that poorly annotated genes encode proteins that perform functions substituting for these missing components. Based on the cluster analysis and the results described below, we propose that ACX60_RS00475 is one such protein that could act as a missing link and have renamed the gene *advA* (antibiotic susceptibility and division protein of *Acinetobacter*). We analyzed *advA* alongside *blhA*, whose phenotypic signature was also connected to chromosome replication-cell division and was extremely similar to that of *advA* (Fig. 2a, b; *r* = 0.94, *p* < 10^−14^).

**Fig. 3:**
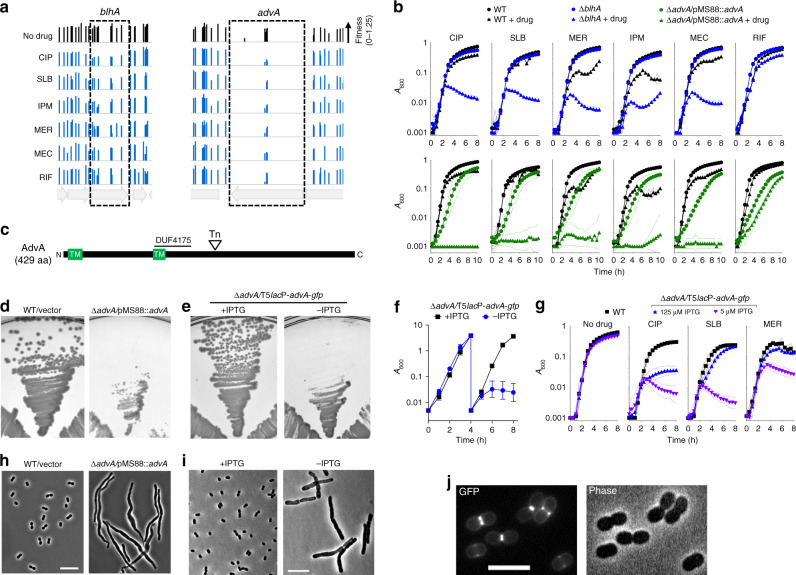
AdvA is a critical cell division protein connected by susceptibility phenotypes to BlhA. **a** *A. baumannii* transposon mutants mapping throughout *blhA* and in a limited region of *advA* show decreased fitness with fluoroquinolones and β-lactams but not RIF. Bars show Tn-seq fitness values of individual Tn*10* mutants at each locus across all tested banks in the indicated condition. **b** Validation of Tn-seq drug susceptibility phenotypes using defined mutants independently cultured in microplates. EGA746 (∆*blhA*, top, blue symbols) or EGA745 (∆*advA*/pMS88::*advA*, bottom, green symbols) were tested in parallel with the same WT control (black symbols). Data are presented as geometric mean (indicated by symbols) ±s.d. (indicated by area-filled dotted bands) from *n* = 3 independent cultures. Where not visible, s.d. is within the confines of the symbol. **c** Schematic of AdvA protein. Tn indicates approximate location of transposons in *advA* analyzed by Tn-seq, TM indicates predicted transmembrane helix. **d**, **e** AdvA is essential for colony formation. EGA745 or control were grown on solid medium at 37 °C (**d**). AFA11 (∆*advA* harboring T5*lac*P-*advA*-*gfp*) was grown on solid medium ± 0.5 mM IPTG (**e**). Images are representative of 18 (**d**) or 3 (**e**) parallel cultures. **f** AdvA is essential for growth in broth. AFA11 pregrown with 1 mM IPTG was diluted into LB ± 1 mM IPTG, followed by dilution into the same medium after 4 h. Growth was monitored by *A*_600_ (1-cm cuvettes). Data points show geometric mean ± s.d. (*n* = 3 independent cultures). **g** AdvA level determines antibiotic susceptibility. AFA11 pregrown with 250 µM IPTG was washed, resuspended in LB with 5 or 125 µM IPTG, and cultured with WT control in microplates with or without the indicated sub-MIC antibiotic (Supplementary Table 1). Data are shown as geometric mean ± s.d. as in (**b**). **h**, **i** *advA* deficiency results in cell filamentation. The indicated strains grown with or without inducer were imaged via phase-contrast. Images are representative of three parallel cultures. Scale bar, 10 µm. **j** AdvA-GFP localizes to mid-cell at sites of cell division. WT *A. baumannii* harboring T5*lac*P-*advA*-*gfp* was cultured to mid-log phase with 50 µM IPTG and imaged by phase-contrast and fluorescence microscopy. Scale bar, 5 µm. Images are representative of two parallel cultures. Source data are provided as a Source Data file.

To show that mutations in *advA* and *blhA* generate the pattern of selective hypersensitivity to fluoroquinolones and β-lactams predicted by Tn-seq for this gene cluster, we constructed in-frame deletions and tested the resulting mutants for growth in broth medium containing antibiotics at concentrations below the MIC determined for WT. The ∆*blhA* mutant had substantial growth defects during challenge with CIP and several β-lactams, but not rifampicin (RIF) (Fig. 3b, blue symbols), in agreement with the relative fitness of *blhA* transposon mutants exposed to the same drugs in our screen (height of boxed bars in Fig. 3a, Supplementary Data 1)^30^. A deletion of *advA*, however, could not be isolated in the absence of a second copy of the gene. Analysis of the location of transposon insertions in *advA* within our Tn-seq banks revealed that they mapped exclusively to a single region corresponding to residues 203–238, downstream of the DUF4175 domain and two predicted transmembrane helices (Fig. 3c), in both Tn*10* (Fig. 3a) or *mariner* pools (Supplementary Fig. 3). These results are consistent with an essential function for *advA*, with only a small subset of transposon insertions in the gene yielding hypomorphic mutants with detectable fitness.

To examine *advA*-associated phenotypes, targeted deletions were isolated in the presence of a complementing DNA fragment (Methods). We used two plasmids for this purpose. The first was a derivative of the R1162*rep*^ts^ Kan^R^ plasmid pMS88^54^ containing a constitutive *advA*. This low copy plasmid shows instability at 42 °C in *E. coli*^54^ and, as described below, is also unstable in *A. baumannii* grown at 37 °C. The second plasmid was a derivative of pEGE305^27^ in which the inducible *lacI*^q^-T5*lac*P module controls expression of an *advA*-*gfp* translational fusion. We found that ∆*advA* cells harboring pMS88-*advA* could not be cured of the plasmid, consistent with essentiality of this gene. To measure efficiency of curing, the strain was restreaked from LB agar plates with kanamycin onto drug-free LB agar, followed by overnight growth at 37 °C. One hundred percent of the colonies from the ∆*advA*/pMS88-*advA* strain retained the plasmid (18/18 retaining Kan^R^); in contrast, pMS88 was lost from a large fraction of the parallel cultured WT control strain (6/18 colonies retaining Kan^R^). In addition, ∆*advA*/pMS88-*advA* showed reduced colony size (Fig. 3d) and delayed growth in liquid medium (Fig. 3b, green vs. black circles) at 37 °C compared to WT. Second, ∆*advA* harboring T5*lac*P::*advA*-*gfp* required IPTG for colony formation (Fig. 3e) and for growth after passage in broth (Fig. 3f). These findings indicate that AdvA is essential for *A. baumannii* growth.

Strikingly, reducing AdvA levels modulated antibiotic susceptibility in the pattern predicted by its phenotypic signature. The ∆*advA*/pMS88-*advA* strain cultured at 37 °C showed selective antibiotic hypersensitivities matching that of ∆*blhA* (Fig. 3b, green symbols). The presence of pMS88 in WT did not affect growth with the same concentrations of fluoroquinolone or β-lactam antibiotics (Supplementary Fig. 3). Moreover, although ∆*advA*/T5*lac*P::*advA*-*gfp* could reach saturation in broth medium with minimal amounts of inducer (5 µM IPTG) in the absence of antibiotics, addition of sub-MIC levels of CIP, SLB, and MER caused substantial growth defects compared to WT, consistent with the Tn-seq results (Fig. 3g). Increasing the inducer concentration to 125 µM enhanced growth with each antibiotic, although CIP susceptibility level was still below that of WT (Fig. 3g). In further support of a role in cell division, both ∆*advA*/pMS88-*advA* and ∆*advA*/T5*lac*P::*advA*-*gfp* after removal of inducer had pronounced filamentous morphologies (Fig. 3h, i), and the AdvA-GFP hybrid localized to mid-cell at sites of ongoing cell division (Fig. 3j). These results together support the predictions of the Tn-seq cluster analysis that *advA* functions in cell division in *A. baumannii* and is a newly identified target for antibiotic hypersensitivity.

### Phenotypic signatures link a cell-wall hydrolase to LOS synthesis

We explored the Tn-seq dataset further to identify phenotypic signatures that predict functions in cell envelope integrity and biogenesis, taking advantage of the diverse types of antibiotic treatments in our screen. We focused first on antibiotics whose action is modulated by OM integrity. The OM impedes the uptake of bulky, hydrophobic antibiotics such as RIF and azithromycin (AZITH), while the OM lipid A component is the target of the amphipathic polymyxins colistin (COL) and polymyxin B (PB)^55^. We predicted that phenotypic signatures defined by hypersusceptibility to these antibiotics would identify proteins that contribute to OM integrity in *A. baumannii*. Principal component analysis (PCA), therefore, was used to identify a set of genes with susceptibility signatures showing dramatic fitness changes as a function of antibiotic hydrophobicity.

**Fig. 4:**
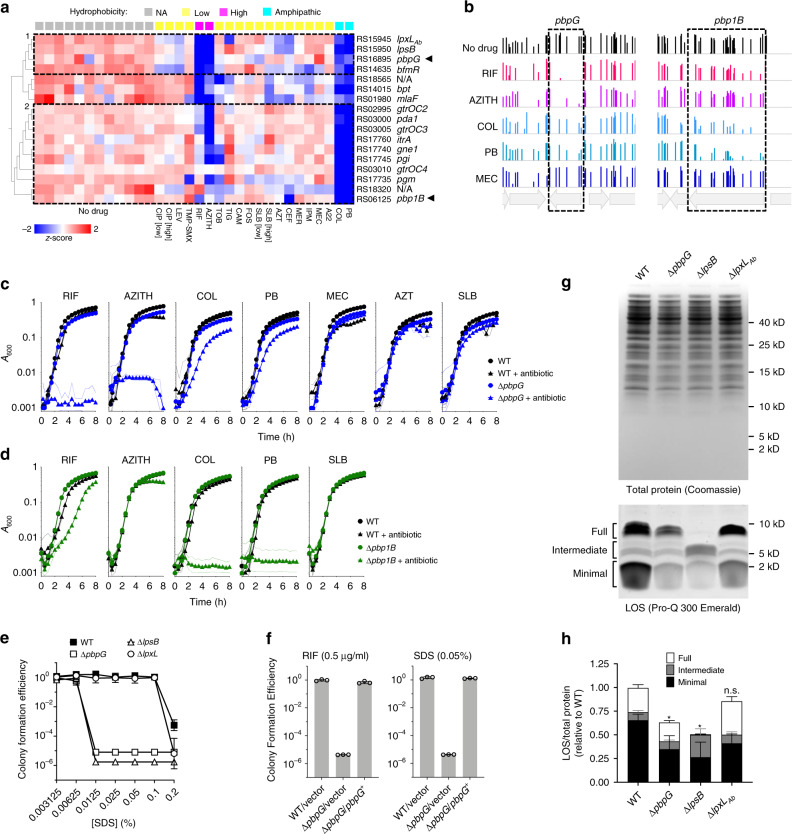
Phenotypic signatures defined by hydrophobic compound sensitivity reveal connection of *pbpG* to LOS synthesis. **a** Fitness profile clusterogram of genes for which knockout causes preferential but differing hypersensitivity to hydrophobic (RIF and AZITH) and amphipathic (COL and PB) antibiotics. Tn-seq data were subjected to PCA to identify discriminating genes whose fitness values differed as a function of hydrophobicity annotation [high, xlogp3 ≥ 4; low, xlogp3 < 4; amphipathic, polymyxin antimicrobial peptides; NA not applicable (no drug); Supplementary Table 1] (ANOVA, *q* = 0.0003). Heatmap shows normalized fitness in *z*-scored units. Dashed boxes indicate clusters of phenotypic signatures defined by differential defects with hydrophobic and amphipathic drugs. Arrowheads highlight cell-wall enzymes clustering with distinct surface polysaccharide synthesis pathways. **b** Bars show fitness values of individual transposon mutants at each locus across all tested banks as in Fig. 3. **c**, **d** Validation of Tn-seq selective drug hypersensitivities using deletion mutants ∆*pbpG* (**c**) or ∆*pbp1B* (**d**) vs. isogenic WT. Data points show geometric mean ± s.d. (*n* = 3 independent cultures, except for WT + MEC, *n* = 2 independent cultures) as in Fig. 3. **e** ∆*pbpG* strain shows detergent hypersensitivity phenotype matching that of *lpsB* LOS glycosyltransferase mutant by colony formation efficiency (CFE) assay. Data points show geometric mean ± s.d. (*n* = 4 independent cultures). **f** Reintroduction of *pbpG* reverses hypersusceptibility to RIF and SDS. Strains harbored vector (pYDE153) or vector containing *pbpG* (pYDE210). Bars show geometric mean CFE ± s.d. (*n* = 3 independent cultures). **g**, **h** *pbpG* knockout results in reduced LOS levels. **g** LOS (bottom) and total protein (top) were detected in cell lysates separated by SDS-PAGE. Image is representative of three experiments. **h** LOS levels in regions indicated in **g** were normalized to total protein content. Values are shown relative to total normalized LOS levels in WT. Bars show mean ± s.d. from *n* = 7 (WT, ∆*pbpG*) or *n* = 5 (*∆lpsB* and *∆lpxL*) biologically independent samples. Total normalized LOS levels of each mutant were compared to WT by two-way ANOVA with Dunnett’s multiple comparisons test. *p < 0.0001; n.s. not significant (*p* = 0.1652). Source data are provided as a Source Data file.

By performing PCA and hierarchical clustering, the resulting signatures could be divided into two general groups based on whether hydrophobic (group 1) or amphipathic character (group 2) was more tightly associated with susceptibility (Fig. 4a, dashed boxes). Hypersensitivity to hydrophobic antibiotics was associated with mutation in *bfmR*, which controls transcription of genes involved in OM synthesis^27^ as well as in three additional genes with highly correlated phenotypic signatures—*lpsB*, *lpxL*_*Ab*_, and *pbpG* (group 1, Fig. 4a, *r* = 0.74–0.82, *p* < 10^−5^). LpsB is a conserved glycosyltransferase critical for LOS core construction. Mutants lacking *lpsB* express a deeply truncated LOS molecule^29^. LpxL_Ab_ is an acetyltransferase responsible for addition of a lauroyl acyl chain to lipid A^28^. *pbpG* encodes a protein related by sequence alignment to *E. coli* PBP7/8, a cell wall D,D-endopeptidase^56^, and by structural homology modeling to D,D-carboxypeptidases^56,57^. *A. baumannii* transposon mutants bearing *pbpG* mutations are attenuated in animal infection models and are complement sensitive^56^, although the contribution of this enzyme to envelope biogenesis is unclear. Group 2 discriminating mutants, showing preferential hypersensitivity to the amphipathic polymyxins, are largely found in OC and K locus genes that determine LOS outer core and capsule biogenesis, respectively^58–60^ (Fig. 4a). Deletion of one of these genes, *itrA*, was shown to cause selective hypersensitivity to COL but not RIF^61^, in agreement with its Tn-seq fitness values. Interestingly, group 2 also included a cell wall synthesis enzyme—the bifunctional transpeptidase/transglycosylase PBP1B (Fig. 4a). Clusters of signatures, therefore, indicate that loss of a subset of peptidoglycan (PG) synthesis enzymes and surface carbohydrate synthesis pathways results in selective hypersensitivity to hydrophobic or amphipathic antibiotics.

To validate these selective changes in antimicrobial susceptibility, we analyzed the effects of targeted, in-frame deletions on growth in the presence of antibiotics (Supplementary Table 1). The ∆*pbpG* mutant showed severe defects with RIF and AZITH, partial defects with COL and PB, and no defect with the β-lactam antibiotics mecillinam (MEC), aztreonam (AZT), and SLB, consistent with its placement in group 1 (Fig. 4b, c). In contrast, ∆*pbp1B* showed severe defects with COL and PB, a partial defect with RIF, and no defect with AZTIH or SLB, consistent with its placement in group 2 (Fig. 4b, d). Interestingly, although it shows similarity to PBP1A which is connected to tolerance of OM defects in *A. baumannii*^62^, the phenotypic signatures of *pbp1A* and *pbp1B* did not correlate (*r* = −0.024, *p* = 0.9), and *pbp1A* mutant bacteria showed no enhanced susceptibility to COL or PB (Supplementary Fig. 4). The role played by *pbpG* in antibiotic resistance was also evaluated in a second *A. baumannii* isolate characterized by multidrug-resistance, AB5075. Two separate AB5075 mutants with different transposon insertions in *pbpG* each showed pronounced growth defects with RIF and AZITH, as well as reduced growth with COL (Supplementary Fig. 4), similar to the corresponding phenotypes in ATCC 17978. Sensitivity to vancomycin (VAN), another antibiotic blocked by the OM, was also increased. In contrast to the situation with ATCC 17978, *pbpG* knockout in AB5075 also enhanced susceptibility to SLB (Supplementary Fig. 4). Therefore, while defense against hydrophobic/bulky and amphipathic antibiotics is a conserved feature linked to *pbpG*, the overall genotype may modulate its relative resistance to other forms of stress.

Given the highly similar pattern of drug sensitivity caused by *pbpG* and LOS core mutations, we examined their connection to maintenance of the OM permeability barrier by comparing their effects on SDS resistance. While WT *A. baumannii* grew efficiently on solid medium containing up to 0.1% SDS, ∆*pbpG* had a pronounced SDS defect and formed colonies only at concentrations of 0.00625% or lower, mimicking the phenotype of ∆*lpsB* (Fig. 4e). By contrast, deletion of *lpxL*_*Ab*_ produced a subtle defect only evident at a high SDS concentration (Fig. 4e). Deficiencies in the two co-clustering LOS synthesis proteins thus have vastly different consequences for the OM barrier. Reintroduction of cloned *pbpG* in the ∆*pbpG* mutant restored both RIF and SDS susceptibility to WT levels (Fig. 4f). The matching hypersensitivity phenotypes caused by knockout of *lpsB* and *pbpG* may reflect related defects in LOS biogenesis.

Analysis of LOS in strains harboring deletions of *pbpG, lpsB*, and/or *lpxL*_*Ab*_ revealed strain-specific defects that show certain common features. Whole-cell lysates from each strain were separated by sodium dodecyl sulphate-polyacrylamide gel electrophoresis (SDS-PAGE) and LOS detected by carbohydrate-specific staining. SDS-PAGE gels were also stained with Coomassie Blue to allow normalization of samples by total protein content (Methods). Consistent with previous observations^29,61^, WT *A. baumannii* LOS was heterogenous with several distinct co-migrating bands ranging from approximately 2–10 kD (Fig. 4g and Supplementary Fig. 4). The LOS banding pattern was not affected by removal of proteins with proteinase K digestion (Supplementary Fig. 4). LOS bands were grouped into three sets that we termed “full,” “intermediate,” and “minimal” based on the hypothesis that degree of glycosylation is a major determinant of band heterogeneity. As expected, the ∆*lpsB* mutant showed an altered banding pattern defined by loss of full-length LOS and accumulation of intermediate forms (Fig. 4g, h). This mutant also had a substantial reduction in overall LOS levels (Fig. 4g, h, sum of all band intensities). *lpxL*_*Ab*_ deletion had a much more subtle effect on LOS banding pattern, with apparent consolidation of some full-length and intermediate bands (Fig. 4g, h). Deletion of *pbpG* resulted in an LOS band pattern appearing similar to WT, but the levels of both full-length and minimal bands were clearly decreased (Fig. 4g, h). The ∆*pbpG* and ∆*lpsB* mutants each showed approximately 40–50% reduction in overall LOS levels compared to WT, in contrast with ∆*lpxL*_*Ab*_ which did not cause overall LOS levels to be significantly altered. Consistent with the different hypersensitivity signatures that separated *pbp1B* mutants from the group 1 cluster, *pbp1B* deletion did not result in appreciable changes in LOS production (Supplementary Fig. 4). The reductions in LOS levels observed with *pbpG* and *lpsB* mutation, which may be the driver of their highly similar hypersensitivity phenotypes, suggest the presence of an unappreciated connection between cell wall and OM biogenesis in *A. baumannii*.

### Phenotypic signature analysis identifies shape determinants

**Fig. 5:**
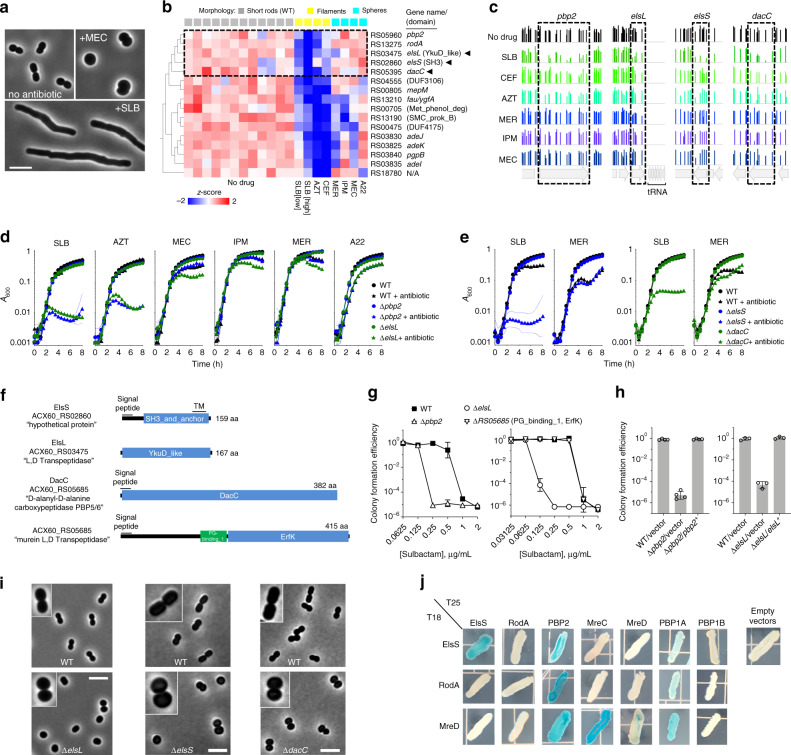
Morphology-specific susceptibility signatures uncover phenotypes and proteins linked to the Rod-system. **a** Exposure of *A. baumannii* to sub-MIC β-lactams causes target-specific morphotypes. SLB (0.25 µg/ml) causes growth as extended rods. CEF and AZT cause a similar filamentous morphology. MEC (16 µg/ml) causes loss of rod shape. IPM and A22 cause a similar spheroid morphology. Images were acquired with phase-contrast. Scale bar, 5 µm. **b** Tn-seq fitness clusterogram showing subset of genes for which inactivation causes selective hypersensitivity to antibiotics generating filaments vs. spheres. Tn-seq data from the indicated conditions were subjected to PCA using morphology annotations (indicated above the heatmap), and discriminating genes having significantly different fitness with cell wall perturbations causing filamentation compared to other conditions were identified (two-sided *t* test, *q* = 0.025). Heatmap shows normalized fitness in *z*-scored units. Dashed box indicates cluster of canonical Rod-system genes with three uncharacterized genes (arrowheads). **c** Fitness values of individual transposon mutants at each locus across all banks as in Fig. 3. **d**, **e** Validation of Tn-seq hypersensitivities using deletion mutants and WT cultured independently. Data points show geometric mean ± s.d. (*n* = 3 independent cultures) as in Fig. 3. **f** Domain predictions in the indicated proteins, listed with NCBI locus tag and protein annotation. ElsL does not contain a predicted signal peptide. **g** SLB susceptibility on solid medium was analyzed via CFE assay. Data points show geometric mean ± s.d. (left, *n* = 3; right, *n* = 4 independent cultures). **h** CFE with SLB (0.25 µg/ml) vs. no drug was measured with strains harboring pEGE305 (vector), pYDE135, (*pbp2*) or pEGE308 (*elsL*). Bars show geometric mean ± s.d. (left, *n* = 4; right, *n* = 3 independent cultures). **i** ElsL, ElsS, and DacC determine Rod shape. Mutants (bottom) and WT control (top) grown without antibiotics to mid-log phase were imaged with phase contrast. Scale bar, 5 µm. Images are representative of 2 (*elsL*), 3 (*elsS*), or 4 (*dacC*) parallel cultures. Insets show 2×-magnified views of representative bacteria. **j** Two-hybrid interactions of ElsS and Rod-system proteins. Proteins fused to a T25 or T18 CyaA fragment were tested in *E. coli* for LacZ reporter activity on X-gal plates. Images are representative of 6 co-transformants patched in parallel. Source data are provided as a Source Data file.

The ability of antibiotics to specifically target distinct aspects of cell wall growth in the Tn-seq screen allowed us to identify new determinants of envelope biogenesis in *A. baumannii*. Cell wall biosynthesis in rod-shaped bacteria is largely governed by two multiprotein machineries, the divisome and the Rod system^63^. The divisome builds PG at the division septum, while the Rod system dictates PG growth along most of the long-axis of elongating bacteria^63^. Different β-lactams typically have distinct affinities for transpeptidase enzymes in each machine, allowing for signature morphological consequences upon drug exposure. For instance, at the sub-MIC doses used in our screen, SLB, AZT, and ceftazidime (CEF) caused *A. baumannii* to abnormally elongate, while MEC, imipenem (IPM), and meropenem (MER) caused cells to become spheres (Fig. 5a)^27^. These morphological changes reflect the described preferences of each β-lactam for transpeptidases acting within the divisome vs. Rod system (divisome > Rod, SLB, AZT, and CEF; Rod > divisome, MEC, IPM, and MER)^64^. The small molecule A22, which inhibits the key Rod-system protein MreB^63^, also produced the expected spherical morphology at sub-MIC. Focusing on the Tn-seq data from these seven treatments and untreated control conditions, we explored the genome via PCA for phenotypic signatures allowing discrimination of the two forms of morphological stress (Fig. 5a). We predicted that the corresponding genes might reveal envelope pathways involved in intrinsic defense against specific block of elongation or division.

A set of discriminating genes was identified whose fitness signatures revealed significant differences between stresses that target the two systems (Fig. 5b). Among these are mutants that showed low Tn-seq fitness with divisome-targeting antibiotics, but relatively high fitness during challenge with Rod-targeting agents (Fig. 5b, dashed box). This cluster included *pbp2* and *rodA*, encoding known members of the Rod system that are nonessential for viability in *A. baumannii* (Supplementary Data 1)^27^. Mutation of *mreB*, *mreC*, and *mreD*, encoding additional key members of the Rod-system, caused a pattern of selective susceptibility across all antibiotics similar to that of *pbp2* and *rodA* (Supplementary Fig. 5, *r* = 0.42–0.82, *p* < 0.018). Targeted deletion of *pbp2* recapitulated the Tn-seq results (Fig. 5b, c), resulting in defective growth with divisome-targeting (SLB and AZT) but not Rod-system targeting (MEC, IPM, MER, and A22) antibiotics in broth culture (Fig. 5d). The MIC of SLB also was lower with ∆*pbp2* compared to WT on solid medium, consistent with the broth results (Fig. 5g). The susceptibility defect with SLB was reversed by *in trans* expression of *pbp2* (Fig. 5h). This result was not dependent on strain background, as a *pbp2* mutation in the multidrug resistant (MDR) background AB5075 resulted in hypersensitivity profiles that were similar to ATCC 17978 (Supplementary Fig. 5). Therefore, when mutations inactivate the Rod system, *A. baumannii* is hypersensitized to β-lactam targeting of the divisome PG synthesis machinery. In contrast, attack by low concentrations of Rod-targeting drugs (MEC, MER, IPM, and A22) on Rod system mutants is indistinguishable from the effects of these treatments on WT.

Strikingly, a gene cluster showing the reciprocal pattern of hypersensitivity, low fitness with Rod-targeting antibiotics, and high fitness with divisome-targeting antibiotics, was not identified. This could be explained by the fact that many proteins of the divisome are essential and corresponding Tn mutants could not be evaluated. These results could also reflect the possibility that Rod complex proteins are able to act within the divisome^63^, while divisome complex proteins cannot act in the Rod complex.

In addition to Rod system members, we identified three uncharacterized genes that co-cluster with mutations in known Rod system-encoding genes and have signatures discriminating between filamentation and sphere-formation (Fig. 5b). The first gene, ACX60_RS03475, encodes a protein with a YkuD-like domain found in L,D-transpeptidase enzymes^65^, with the others encoding an SH3_and_anchor domain (ACX60_RS02860) and a protein with homology to PBP5 and PBP6 D,D-carboxypeptidases (ACX60_RS04555, DacC^66^) (Fig. 5f). The susceptibility signatures of these three genes, which were defined by hypersensitivity to SLB but not antibiotics targeting the Rod system, are significantly correlated with those of Rod system mutants (Fig. 5c and Supplementary Fig. 5, *r* = 0.44–0.78, *p* < 0.011). It is likely that the products of these genes are necessary for Rod system function. Based on their phenotypic signatures and the experiments described below, we have named ACX60_RS03475 *elsL* and ACX60_RS02860 *elsS* (elongation and SLB susceptibility defects, containing L,D-transpeptidase family catalytic domain or SH3 domain, respectively).

We pursued *elsL*, *elsS*, and *dacC* in subsequent analyses with targeted deletions. Each deletion resulted in selective susceptibilities to divisome-targeting but not Rod system-targeting antibiotics, with defects mimicking those caused by ∆*pbp2* (Fig. 5d, e, g). In the presence of low levels of SLB, the *∆elsL* growth defect was reversed by reintroducing the cloned gene (Fig. 5h). The specificity of this result is emphasized by the fact that deletion of a second predicted L,D-transpeptidase (ACX60_RS05685, Fig. 5f) had no effect on SLB susceptibility (Fig. 5g and Supplementary Fig. 5), consistent with the lack of effects in Tn-seq fitness challenge with most antibiotics (Supplementary Fig. 5). Therefore, *elsL* likely plays a dominant role in modulating β-lactam susceptibility. The mutation in *elsL* also caused a severe and selective growth defect with SLB in the MDR AB5075 background (Supplementary Fig. 5).

Loss of key Rod-system proteins causes characteristically rod-shaped cells to form spheroids^27^. Given the phenotypic signatures that connect *elsL*, *elsS*, and *dacC* with the Rod system, we predicted similar phenotypes with mutations in these genes. Indeed, deletion of *elsL*, *elsS*, or *dacC*, but not ACX60_RS05685, caused cells to lose rod shape and become spherical (Fig. 5i; Supplementary Fig. 5), mimicking the effect of antibiotics that block the Rod system^27^ (Fig. 5a). *elsL* mutation also caused the MDR strain AB5075 cells to become spherical, indicating that the encoded protein functions similarly in recent clinical isolates (Supplementary Fig. 5).

The specific morphological and antibiotic susceptibility changes in *elsL*, *elsS*, and *dacC* mutants matching those caused by Rod-system block could be explained in at least two ways: the mutations could cause defects that indirectly affect Rod system function, or the encoded proteins could themselves be important components of the Rod complex. We considered ElsS a candidate for the latter, based on analogy with *Helicobacter pylori*, in which an SH3 domain protein serves to scaffold a cell wall synthesis complex^67^. To determine protein interactions with ElsS, we fused its predicted soluble C-terminus (Fig. 5f) to fragments of adenylate cyclase (CyaA) for analysis using the bacterial adenylate cyclase two-hybrid system^68^. The ElsS chimeras were used to probe its interactions in *E. coli* with Rod system proteins fused to a complementary CyaA fragment (Methods). Interactions were found among the *A. baumannii* orthologs of Rod complex members, including PBP2, RodA, MreC, MreD, and PBP1A (Fig. 5j). In addition, ElsS generated a two-hybrid readout consistent with homo-oligomerization and interaction with at least one key Rod system component, PBP2 (Fig. 5j). A weak two-hybrid signal also resulted between ElsS and PBP1A, but not PBP1B, which is a member of the division complex^63^ (Fig. 5j). Clustering of phenotypic signatures has therefore identified a novel shape determinant with the potential to directly modulate the *A. baumannii* PG assembly machinery.

### Tn-seq analysis predicts synergistic antimicrobial combinations

**Fig. 6:**
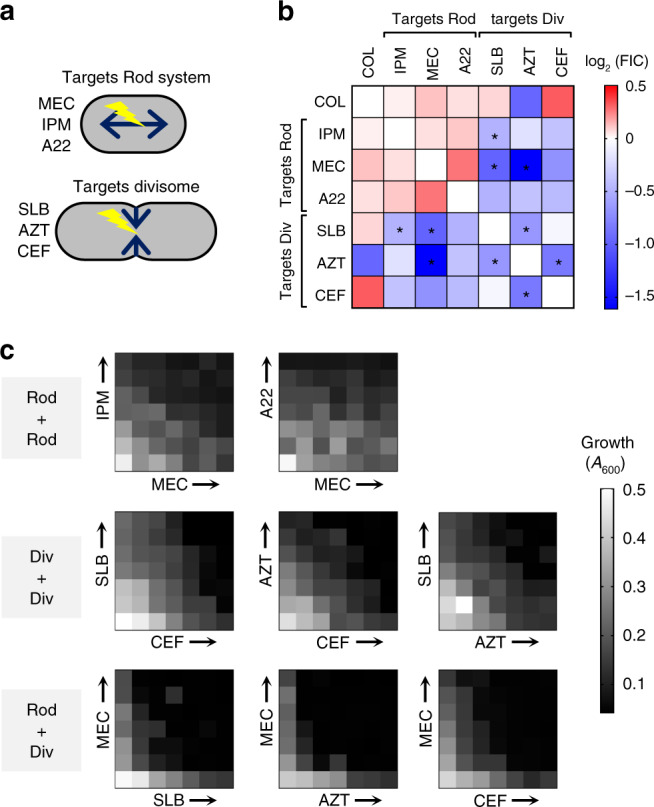
Synergistic inhibition of *A. baumannii* by pairing Rod-system-targeting and Divisome-targeting antibiotics. **a** Diagrams showing the two modes of cell wall growth in rod-shaped bacteria which are governed by distinct biosynthesis systems (Rod-system vs. divisome). Listed next to each diagram is the subset of antibiotics that preferentially target the respective PG synthesis system. **b** Heat map shows average Log2FIC scores resulting from pairwise interactions among seven antibiotics via the diagonal sampling method from *n* = 2 independent determinations. Blue indicates synergistic pairs, white indicates additive pairs, and red indicates antagonistic pairs. *Average Log2FIC was significantly different from 0 in one-sample *t* test (two-tailed *p* < 0.05; exact *p* values shown in Supplementary Table 3). **c** Validation of drug–drug interactions via checkerboard assay. Heat map shows bacterial growth in microplate wells containing no drug (lower left wells) or increasing amounts of each drug alone or in pairwise combinations. Drug concentrations increase linearly from left to right along *x*-axis, and bottom to top along *y*-axis. “Div” refers to Divisome, “Rod” refers to Rod-system. Source data are provided as a Source Data file.

On the basis of the result that mutational block of the Rod-system sensitizes *A. baumannii* to antibiotics attacking divisome PG synthesis, we hypothesized that combining an antimicrobial that targets the Rod-system with one that targets the divisome would achieve synergistic killing. Pairwise combinations of antibiotics targeting each system (Fig. 6a) were systematically tested for ability to block bacterial growth using an established method (diagonal sampling) that allows high numbers of drug interactions to be tested in parallel^69^. COL, a drug used in some combination therapies targeting Gram-negatives^70^, was also included in interaction testing. The log2-transformed fractional inhibitory concentration (FIC) was used to quantify drug interactions^71^. Reminiscent of our results showing that simultaneous mutational block and antibiotic targeting of the Rod system fails to generate synergistic growth defects (Fig. 5b–e), simultaneous block of Rod system function by two agents showed an absence of synergistic effects (Fig. 6b, Supplementary Table 3). By contrast, when a Rod-targeting agent was combined with a divisome blocker, a log2FIC value < 0 was seen in every pairing (Fig. 6b, Supplementary Table 3), consistent with a synergistic interaction. Checkerboard assays confirmed these results and were again consistent with strong synergism as addition of divisome blocking drugs (AZT, CEF, or SLB) with a Rod-targeting drug (MEC) showed strong synergy (Fig. 6c). This mimicked the consequences of adding divisome-blocking drugs to mutants defective in Rod system function (Fig. 5b, d). In pairings of two divisome-targeting agents, those involving AZT also showed negative log2FICs, and checkerboard tests confirmed modest synergy (Fig. 6c, Supplementary Table 3). Pairings with COL showed a mix of positive and negative log2FIC values, none of which was significantly altered from log2FIC 0 (Fig. 6b, Supplementary Table 3).

## Discussion

In these studies, we have systematically analyzed determinants of drug susceptibility in *A. baumannii*. Many of these determinants are encoded by nonessential genes that become essential during antibiotic therapy, allowing the identification of novel targets for potentiating antibiotics that have lost potency against the pathogen. The high saturation of Tn-seq insertions also allowed analysis of position-specific hypomorphic alleles in essential genes, such as *advA*. In addition, several genes showed increased fitness during antibiotic challenge when mutated, identifying pathways that may contribute to evolution of enhanced antibiotic resistance. By examining how arrays of susceptibility phenotypes across diverse antibiotics are linked within the genome, we generated new leads on the functions of a variety of poorly characterized genes in multiple facets of envelope biogenesis.

Understanding the idiosyncrasies of envelope synthesis in *A. baumannii* can provide a path to attack the pathogen specifically. The organism has diverged from the Gram-negative paradigm and conspicuously lacks canonical proteins that coordinate cell septum formation with chromosome replication (ZapB and SlmA) and cell separation (FtsE/X)^24^. Interestingly, cell division defects such as those caused by knockout of FtsZ-associated proteins or BlhA cause hypersensitivity to antibiotics targeting cell wall and DNA synthesis in *A. baumannii*^30^. Therefore, identifying susceptibility signatures in this fashion is likely to be an effective strategy of identifying “missing link” factors involved in coordinating cell division with DNA synthesis. Through this strategy, we identified a previously uncharacterized gene, *advA*, whose phenotypic signature in response to antibiotic stress strongly correlated with *blhA* and genes coordinating chromosome replication and cell division. AdvA was shown to localize to cell division sites, while its depletion caused a lethal filamentation phenotype, consistent with a critical role in cell division (Fig. 3h, i). Based on this functional analysis and its phenotypic signature relationships, AdvA may represent a key component of a chromosome-divisome coordination system that substitutes for, or bypasses the need of, ZapB and/or SlmA. Given the close correlation of its phenotypic signature with *advA* and its less extreme growth phenotype, *blhA* may encode a nonessential component or modulator of the process controlled by AdvA. As with BlhA^30^, sequence alignment yielded little insight on the function of AdvA, but structural modeling^57^ predicted that its N-terminal region assumes a fold resembling the sensor domain of two-component system kinases, albeit of low sequence identity (Supplementary Fig. 3). Work is ongoing to dissect the role of this protein in coordinating cell division.

We leveraged the diversity in both subcellular targets and physiochemical properties of our tested antibiotics to mine the Tn-seq susceptibility signatures for unappreciated factors enhancing envelope resiliency. This led to the surprising result that a predicted cell wall hydrolytic enzyme, encoded by *pbpG*, is required to maintain integrity of the OM permeability barrier. Defects in PbpG and in the LpsB core LOS glycosyltransferase each reduce overall LOS synthesis to similar levels (sum of band intensities in Fig. 4h). Inefficient LOS production coupled to either lesion may allow phospholipids to accumulate at higher density in the OM outer leaflet, weakening the barrier against lipophilic compounds^55^. The LOS defect also explains the impressive virulence attenuation of *pbpG* mutants^56^. How *overall* LOS synthesis is promoted by LpsB is not clear and is particularly puzzling with PbpG, a cell wall enzyme. One possible model is that defects in either protein results in altered LOS transit, triggering a down-shift in general production of LOS and possibly other OM components by a sensory system^72^. In *E. coli*, “deep rough” mutants akin to *lpsB* produce periplasmic LPS products that are implicated in such a response^73,74^. In the case of PbpG, we hypothesize that the enzyme is the PG hydrolase allowing passage of bulky LOS through the cell wall by the Lpt complex in *A. baumannii*^75,76^. In this model, *pbpG* mutation would indirectly lead to increased periplasmic LOS resulting from this block in transit. Intriguingly, PBP1B was also implicated in maintenance of OM integrity, with mutations in this enzyme causing selective polymyxin susceptibility that resembles the phenotypes of K or OC locus mutations. These findings together reveal additional ways that PG and OM synthesis pathways are tightly intertwined in *A. baumannii*^62^ and indicate that targeting the cell wall may potentiate both antibiotic permeation and immune attack against these pathogens.

One of the most striking results from this work was the ability to predict synergistic relationships between β-lactam antibiotics based on antibiotic hypersensitivity of Tn-seq mutations (Figs. 5 and 6). Key to this approach was demonstrating that cell wall-disrupting antibiotics caused distinct morphological defects in *A. baumannii* that were dependent on the identity of their specific targets. Antibiotics that disrupt cell wall elongation (Rod-system targeting), such as MEC and IPM, were shown to form rounded cells, while divisome targeting antibiotics, such as AZT, resulted in filamentous forms (Fig. 5a)^27,64^. Sensitivity to divisome-targeting drugs was clearly potentiated by mutations affecting elongation, while the identical mutations had little effect on fitness during treatment with Rod-system targeting drugs (Fig. 5b). As mutations in the Rod system potentiate the action of divisome-targeting drugs and generate morphological forms that phenocopy sphere-generating drugs, we reasoned that drugs that induce filament and sphere morphologies should synergize with each other. In fact, AZT (filaments) and MEC (spheres) strongly synergized to kill *A. baumannii*, as predicted by the genetic analysis, whereas rod-targeting pairs such as IPM/MEC revealed no such effects (Fig. 6). This demonstrates that antibiotic synergies can be identified between drugs that target a single bacterial cell structure if the downstream consequences of each treatment can be morphologically distinguished. Our data agree with a strategy involving MEC described in *E. coli*^77^, and support the hypothesized mechanism by which diazabicyclooctanone adjuvants potentiate certain β-lactams against MDR strains of *A. baumannii* and *P. aeruginosa*^78,79^. It should be noted that the method of cytological profiling of bacterial cells in response to antibiotics has recently been shown to differentiate two cell wall-acting antibiotics from each other based on morphotypes^80^. Adding distinguishing variables within antibiotic classes to strategies that involve cytological profiling could be an important tool in developing new antimicrobials or identifying new strategies of combinatorial therapy. Similarly, relationships between different antibiotics based on discriminating variables across the genome has the potential to allow a more complete understanding of the mechanism of action of incompletely characterized antibacterial compounds^18^.

Our overall approach should allow drug class synergies to be predicted as well as drive the identification of new drug targets that could potentiate currently available antimicrobials. For instance, the phenotypic signature of Rod system mutants permitted discovery of previously unrecognized elongation-determining proteins in addition to showing synergy between β-lactams. The identification of these new proteins not only gives insight into mechanisms of PG growth that are specific to *A. baumannii*, but also identifies an attractive physiological process that could be targeted for designing new drugs. Similarly, clustered relationships that define mutations with similar phenotypes across drug classes allowed the identification of new candidate cell division proteins, at least one of which (AdvA) appears essential for *A. baumannii* growth. The identification of pathogen-specific proteins in essential physiological processes is an excellent first step in the development of designer drug therapies that allow specialized targeting of a subset of pathogens. To take full advantage of this strategy, however, drug hypersensitivity approaches must be developed that directly target the subset of essential genes shared by *A. baumannii* clinical isolates. We are currently developing these approaches in order to have a coordinated attack on the central essential physiological processes that support the survival and growth of this emerging pathogen.

## Methods

### Bacterial strains, growth conditions, and antibiotics

Bacterial strains used in this work are described in Supplementary Table 4. *A. baumannii* strains were derivatives of ATCC 17978 unless otherwise stated. Bacterial cultures were grown at 37 °C in Lysogeny Broth (LB) (10 g/L tryptone, 5 g/L yeast extract, 10 g/L NaCl) with aeration in flasks by shaking or in tubes on a roller drum. Growth was monitored by measuring absorbance at 600 nm via spectrophotometer. LB agar was supplemented with antibiotics (ampicillin, 50–100 μg/ml; carbenicillin, 50–100 μg/ml; chloramphenicol (CAM), 25 µg/ml; gentamicin, 10 µg/ml; kanamycin, 10–25 μg/ml; tetracycline, 10 µg/ml; or sucrose, 10%) for strain isolation as needed.

### Molecular cloning and isolation of defined mutants

Plasmids used here are listed in Supplementary Table 4. DNA fragments were amplified using oligonucleotide primers (IDT, Supplementary Table 5) and were usually cloned in pUC18 before subcloning to vectors for recombination or gene expression. Gene deletions were constructed by three-way ligation of ~1 kb flanking homology arms with pSR47S or pJB4648. Deletions of *advA*, *blhA*, *pbpG*, *lpsB*, *lpxL*_*Ab*_, ACX60_RS05685, *dacC*, and *elsS* were constructed in-frame. ∆*elsL* was constructed as a deletion of the first 75 codons via a 500 bp 3′ homology arm due to difficulty cloning a homology arm extending into downstream tRNA sequences (Fig. 5c). *A. baumannii* ATCC 17978 was electroporated with deletion constructs and allelic exchange mutants were isolated via homologous recombination with two selection steps^61^. ∆*advA* was isolated by transforming *advA*^WT^/∆*advA* merodiploids with plasmids containing complementing DNA fragments (pEGE292 or pEGE309; Supplementary Table 4), followed by sucrose counterselection and screening for CIP^S^ ∆*advA* double recombinants. In the case of pEGE292, all steps were carried out at temperatures at or below 30 °C. In the case of pEGE309, double recombinants were isolated in the presence of 1 mM IPTG. Isolation of deletion mutants was verified by colony PCR.

A constitutive *advA* was constructed by cloning *advA* including 78 bp upstream sequence into the HincII site of pUC18 such that the ORF start site was oriented proximal to the PstI site. After digestion with PstI and XbaI, the resulting *advA* fragment was subcloned into the PstI and NheI sites of pMS88 to generate pEGE292. An *advA*-*gfp* translational fusion was constructed by PCR-amplifying an *advA* fragment using primers incorporating an upstream BamHI site and an in-frame XbaI site replacing the stop codon. This site was ligated to a fragment containing *gfp* with an in-frame XbaI site and downstream PstI site cloned in pUC18. The *advA*-*gfp* construct was subcloned into pEGE305 downstream of T5*lac*P via EcoRI and PstI sites to generate pEGE309. *elsL* and *pbp2* were cloned into pEGE305 using the same sites. *pbpG* was cloned into a derivative of pEGE305 (pYDE153) containing an expanded multiple cloning site (Supplementary Table 4).

AB5075-UW and defined T26 transposon insertion mutants were obtained from the Manoil lab three-allele collection^81^. Each mutant was purified from single colonies on LB plates. Transposon location and absence of predicted second-site mutations was determined by whole-genome resequencing via modified small-volume Nextera method and BRESEQ^27,82^ and by screening on Tc plates. Two independent AB5075 transposants for *pbpG* and *elsL* and one for *pbp2* were analyzed.

### Transposon mutant libraries

Tn*10* (altered target specificity) mutant banks constructed in *A. baumannii* ATCC 17978 with plasmid pDL1073^20^ were used with most Tn-seq experiments. Tn-seq experiments with LEV and TMP-SMX employed *mariner* mutant banks constructed in ATCC 17978 using pDL1100. pDL1100 contains a Kan^R^ *mariner* derivative, a hyperactive C9 mutant *mariner* transposase gene downstream of the phage lambda P_L_ promoter, a pSC101ts origin of replication, and a CAM resistance gene (Supplementary Fig. 6). Tn libraries were isolated by electroporation of 50 µl washed cells with pDL1073 (100 ng) or pDL1100 (200–300 ng) in a 0.1-cm-gap-length cuvette via Gene Pulser (Bio-Rad) at 200 Ω, 25 µF, and 1.8 kV. Electroporated cells were diluted with 1 ml SOC broth and spread onto membrane filters (0.45 μm pore size) overlaid on prewarmed LB agar plates either immediately (pDL1073) or after 15 min incubation with the SOC broth at 37 °C (pDL1100). After incubation on filters at 37 °C for 2 h (pDL1073) or 1 h (pDL1100), the filter membranes were transferred to prewarmed LB agar plates containing 20 µg/ml Km. Colonies arising after overnight incubation at 37 °C were lifted from filters by agitation in sterile PBS, combined with glycerol (10% v/v), aliquoted and stored at −80 °C.

### Transposon library antibiotic challenge

Aliquots of transposon mutant banks (each containing approximately 5000–20,000 random mutants) were cultured in parallel in 10 mL liquid LB medium at 37 °C without or with graded concentrations of antibiotics from *A*_600_ 0.003 (*t*_1_) to *A*_600_ 0.5–1 (*t*_2_)^20^. Samples taken from both time points were stored at −20 °C. With most drug treatments, *t*_2_ was approximately 1 h after the *t*_2_ of untreated control to allow both control and treated cultures to reach similar final density and growth phase. Drug-treated samples that showed 20–30% inhibition of growth rate relative to untreated control were chosen for analysis. In most cases, slightly different antibiotic concentrations yielded the optimal 20–30% inhibition with different independent libraries on different days, resulting in the binned concentration ranges across biological replicates shown in Supplementary Table 1. Ten independent transposon libraries were analyzed with each antibiotic treatment. Most drug treatments were performed in pairs with a single untreated control, resulting in 20 distinct treatment conditions and 12 independent untreated controls. Average population expansions between *t*_1_ and *t*_2_ with each condition based on *A*_600_ measurements are listed in Supplementary Data 1.

### Tn-seq Illumina library preparation and fitness calculations

Illumina sequencing libraries were prepared using a modified Nextera method^20^. Genomic DNA (30 ng) was tagmented in a 10 µl reaction mixture at 55 °C for 5 min, followed by inactivation at 95 °C for 0.5 min. 40 µl of PCR master mix containing primers olj928 (for Tn*10* libraries) or olj638 (for *mariner* libraries) and Nextera 2A-R (0.6 µM final) and NEB Q5 high-fidelity polymerase (primers listed in Supplementary Table 5) was added to amplify transposon-adjacent DNA. Reaction conditions were 98 °C for 10 s, 65 °C for 20 s, and 72 °C for 1 min (30 cycles), followed by 72 °C for 2 min. 0.5 µl of this PCR was used in a second PCR containing nested indexed primers (Left Tn*10* or *mariner*-specific indexing primer, 0.6 µM), right indexing primer (0.6 µM), and Q5 polymerase in 50 µl total volume. The reaction conditions were 98 °C for 10 s, 65 °C for 20 s, and 72 °C for 1 min (12 cycles), followed by 72 °C for 2 min. A 9 µl sample was separated on a 2% agarose–Tris-acetate-EDTA (TAE) gel containing SYBR Safe (Invitrogen), and image intensity in the 250–600 bp region was used to quantify and pool each PCR product in equimolar amounts. The multiplexed libraries were purified on a Qiagen QIAquick column, and 15–20 pmol DNA was used in a 50 µl reconditioning reaction with primers P1 and P2 (0.6 µM) and Q5 polymerase. The reaction conditions were 95 °C for 1 min, 0.1 °C/s ramp to 64 °C, 64 °C for 20 s, and 72 °C for 10 min. After purification (Qiagen QIAquick kit), multiplexed libraries were quantified, size-selected (250–600 bp; Pippin HT), and sequenced (single-end 50-bp) by the Tufts University Genomics Core Facility. Sequencing was with an Illumina HiSeq 2500 with high-output V4 chemistry and custom primer olk115 (Tn*10* libraries) or mar512 (*mariner* libraries).

Sequencing read data were processed (BBDuk and FASTX-toolkit), mapped to chromosome (NZ_CP012004) and pAB3 (NZ_CP012005) (Bowtie 1.1.2), and used to calculate individual transposon mutant fitness using our published pipeline^20,83^. The fitness of an individual mutant (*W*_i_) is calculated based on mutant vs population-wide expansion between t_1_ and t_2_ using Eq. (1)^84^:

1 $$W_{\mathrm{i}} = \frac{{{\mathrm{ln}}\left( {N_{\mathrm{i}}\left( {t_2} \right) \times d/N_{\mathrm{i}}\left( {t_1} \right)} \right)}}{{{\mathrm{ln}}\left( {\left( {1 - N_{\mathrm{i}}\left( {t_2} \right)} \right) \times d/\left( {1 - N_{\mathrm{i}}\left( {t_1} \right)} \right)} \right)}},$$

in which *N*_i_(*t*_1_) and *N*_i_(*t*_2_) are the mutant frequency at *t*_1_ and *t*_2_, respectively, and *d* is the population expansion. For a given treatment condition, gene-level fitness was calculated by averaging the fitness of all transposon mutants (across all mutant pools) having insertions in the first 90% of the gene. These fitness scores were normalized to the fitness assigned to 18 “neutral” genes (pseudogenes or endogenous transposon-related genes) throughout the genome to enhance the accuracy of relative fitness measurements across diverse conditions^20^. With TOB treatment, LOWESS curve fitting for fitness normalization was performed via Prism 8 (Graphpad). For each antibiotic, difference in gene-level fitness between treatment and control conditions was deemed significant if fitness was calculated from *n* ≥ 3 individual mutants, magnitude of difference was >10%, and *q*-value was <0.05 (unpaired, two-tailed *t* test with FDR controlled by the two-stage step-up method of Benjamini, Krieger, and Yekutieli, Prism 8)^20^. Fitness scores per insertion along a genomic region were visualized with Integrative Genomics Viewer^85^ after aggregating all scores across multiple independent transposon mutant libraries via the SingleFitness script^83^.

### Phenotypic signature analysis

Hierarchical clustering of phenotypic signatures (gene-level fitness values compiled across all conditions) was performed by average linkage method using Qlucore Omics Explorer (3.5) and Cluster 3.0^86^ and shown as dendrograms. Pearson correlation (*r*) matrices were displayed as heatmap in Prism 8. Identification of discriminating phenotypic signatures by PCA was performed by using Qlucore Omics Explorer (3.5). After prefiltering out essential genes showing low (<0.11) fitness in untreated samples, fitness data were centered and scaled to zero mean and unit variance. Variables with low overall variance were filtered out, and PCA was used to visualize the data in three-dimensional space. Two-group or multigroup statistical testing was used to determine the significance with which variables could discriminate between annotated conditions. *P* values were adjusted for multiple testing (*q*-value) using the Benjamini–Hochberg method, and discriminating variables with *q*-values below the indicated cut-off, resulting in 16–17 variables, were subjected to hierarchical cluster analysis.

Functional information determined from homology on ATCC 17978 gene products was obtained by querying their NCBI protein IDs with UniProt and KEGG Automatic Annotation Server (Supplementary Data 3). Entries matching the query with 100% identity were used to determine gene names, protein descriptions, functional categories, and Gene Ontology terms. These annotations, along with cited experimental evidence if available, were used to place well-annotated genes into shared functional pathways for targeted phenotypic signature analysis. In the case of periplasmic proteases and adapter protein, annotations were aided by direct BLASTp search of ATCC 17978 proteins for orthologs of the characterized *Pseudomonas aeruginosa* proteins. In regard to proteins with limited functional information that arose from genome-wide analysis of phenotypic signatures, we determined protein domains using conserved domain database (CDD)^46^, predicted topology using SignalP-5.0^87^, Phobius^49^, and CCTOP^88^, and structural homology using Phyre2^57^.

### Validation of antibiotic susceptibilities identified by Tn-seq

Pure cultures of defined mutants were diluted to *A*_600_ 0.003 and grown ± antibiotic in 96-well microplate format at 37 °C with shaking in a plate reader (Tecan M200 Pro using iControl; Biotek Epoch 2, or Biotek Synergy H2M using Gen5 software). Growth was monitored by recording *A*_600_, and data exported as blank-corrected values using Megellan or Gen5 software. Antibiotic concentrations used are listed in Supplementary Table 1 unless otherwise noted. To measure sensitivity to SDS, RIF, and SLB by the colony formation efficiency (CFE) assay^27^, serial dilutions of WT and isogenic deletion mutants were grown in absence or presence of graded concentrations of SDS or antibiotic on solid LB agar medium. After overnight growth at 37 °C colony formation was enumerated and compared to untreated control. Limit of detection was approximately 10^−5^–10^−6^.

### LOS analysis

Bacteria were cultured to *A*_600_ ~0.5. One milliltre was harvested by centrifugation, washed with PBS, then repelleted and resuspended in a volume of 1× Novex Tricine SDS sample buffer (Invitrogen) normalized for cell density (50 µl per 1 ml *A*_600_ 0.5). Samples were boiled for 15 min and either cooled on ice (no proteinase K) or incubated with proteinase K (NEB) at 55 °C for 1 h. Samples were re-boiled and electrophoresed using the tricine buffer system with Novex tricine 16%-acrylamide gels (Invitrogen). Spectra Multicolor Low Range Protein Ladder (Thermo) was included to indicate approximate molecular weights. Gels were fixed, washed, stained using Pro-Q Emerald 300 (Invitrogen), and imaged using UV transillumination (Biorad Chemidoc MP). Gels were subsequently stained with Coomassie Brilliant Blue for detection of total protein. Image lab software (Biorad) was used to quantify LOS or total protein intensity levels. Samples were normalized by dividing the LOS intensity level of each band region by the total protein level from Coomassie staining. Relative values were calculated by dividing each normalized LOS value by the total normalized LOS levels in WT.

### Microscopy

Bacteria were immobilized on agarose pads (1% in PBS), and imaged via 100x/1.3 phase-contrast objectives on a Zeiss Axiovert 200 m using OpenLab software, or Leica AF6000 microscope using LAS X (Leica) software, with fluorescence detection using GFP filter cube.

### Bacterial two-hybrid analysis

ElsS, MreD, and RodA hybrids were constructed by fusing the protein’s C-terminus to the N-terminus of the CyaA fragment in pUT18 and pKNT25. Pbp2, MreC, PBP1A, and PBP1B hybrids were constructed by fusing the protein’s N-terminus to the C-terminus of the CyaA fragment in pKT25 (Supplementary Tables 4 and 5). Plasmids encoding CyaA fusions were cloned using XL1-blue at 30 °C. Two-hybrid plasmid pairs were then co-transformed into BTH101 (*cya*-99). Transformants were isolated on LB agar plates containing carbenicillin and kanamycin at 30 °C. Transformants were patched on LB agar indicator plates containing the same antibiotics plus IPTG (0.5 mM) and X-gal (40 μg/ml). Plates were incubated at 30 °C for 24–48 h and imaged with darkfield illumination.

### Drug interaction assays

Drug interaction experiments were performed in 384-well plates. Drugs were printed via a digital drug dispenser (D300e Digital Dispenser, HP) using randomized dispense locations to minimize plate position effects. Bacterial growth was determined by measuring A_600_ after 16 h at 37 °C without shaking (BioTek Synergy HT). Plate measurement data were reconstituted and analyzed (MATLAB, Mathworks) by the diagonal sampling method to determine FIC values from seven drug–drug interactions^69,71^. Bacterial sensitivity to linearly increasing drug dose up to MIC was determined for each single drug and each pairwise two-drug mixture, and FIC values were calculated by comparing sensitivity to the drug mixture with sensitivity to each single drug. Checkerboard assay was used to validate interactions and were quantified using the alpha scoring method^89^.

### Statistics and reproducibility

Statistical analyses of aggregated per-gene Tn-seq mutant fitness data was performed using Perl 5 (version 18). For identifying discriminating variables from phenotypic signature data, statistical tests were performed using Qlucore Omics Explorer (3.5). All other statistical and graphical data analyses were performed using GraphPad Prism 8. Differences between per-gene fitness scores were analyzed by multiple unpaired, two-tailed *t* tests with FDR controlled by two-stage step-up method of Benjamini, Krieger, and Yekutieli. Phenotypic signature correlations were computed as Pearson correlation coefficients with two-tailed *p* values. LOS levels were analyzed by two-way ANOVA with Dunnett’s multiple comparisons test. Log2FIC scores from drug interaction tests were analyzed by two-tailed one-sample *t* test. Experiments were performed at least twice independently with similar results, except drug–drug interaction checkerboard analysis, which were performed once as confirmation of diagonal sampling assay results. Experiments with defined mutants used at least three biologically independent replicates except where noted in the figure legends. Tn-seq experiments used at least ten separate transposon pools cultured independently.

### Reporting summary

Further information on research design is available in the Nature Research Reporting Summary linked to this article.

## Supplementary information

Supplementary Information

Description of Additional Supplementary Files

Supplementary Data 1

Supplementary Data 2

Supplementary Data 3

Reporting Summary

## Data Availability

Source data are provided with this paper. All sequence data can be found in the NCBI Sequence Read Archive under accession codes: PRJNA485788, PRJNA485840, PRJNA486258, PRJNA486803, PRJNA488082, PRJNA636211, PRJNA637031, PRJNA638415, PRJNA638316, PRJNA639060, PRJNA638887, PRJNA485590. Publicly available protein functional information databases used were UniProt and KEGG. Additional datasets are present in the article and Supplementary Information files. All other data and all genetic material used for this paper are available from the authors on reasonable request.

## Source data

Source Data

## Code availability

Scripts used for sequencing read analysis can be found at https://github.com/jsa-aerial/aerobio and https://github.com/vanOpijnenLab/MAGenTA.
