# Author Correction: Antibiotic susceptibility signatures identify potential antimicrobial targets in the *Acinetobacter baumannii* cell envelope

**DOI:** 10.1038/s41467-020-20098-z

**Published:** 2020-11-24

**Authors:** Edward Geisinger, Nadav J. Mortman, Yunfei Dai, Murat Cokol, Sapna Syal, Andrew Farinha, Delaney G. Fisher, Amy Y. Tang, David W. Lazinski, Stephen Wood, Jon Anthony, Tim van Opijnen, Ralph R. Isberg

**Affiliations:** 1 Department of Biology, Northeastern University, Boston, MA 02115 USA; 2 Department of Molecular Biology and Microbiology, Tufts University School of Medicine, Boston, MA 02111 USA; 3 Laboratory of Systems Pharmacology, Harvard Medical School, Boston, MA 02115 USA; 4 Biology Department, Boston College, Chestnut Hill, MA 02467 USA

**Keywords:** DNA replication, Antimicrobials, Cellular microbiology, Microbial genetics

Correction to: *Nature Communications* 10.1038/s41467-020-18301-2, published online 9 September 2020

The original version of this Article contained the following errors:

In the Results, subsection ‘Phenotypic signature analysis identifies shape determinants’, a protein with homology to PBP5 and PBP6 D,D-carboxypeptidases was incorrectly referred to as ‘ACX60_RS04555’; the protein name has been corrected to ‘ACX60_ RS05395’.

Another mislabelling error was present in Figure 5f; the correct version states ‘ACX60_ RS05395’ in place of ‘ACX60_RS05685’.

In addition, the colour fill within dotted error lines (indicating s.d.) was missing from Figures 3b, 3g, 4c, 4d, 5d and 5e.

These errors have been corrected in both the PDF and HTML versions of the Article.

