# Supplementary material for: Antibiotic susceptibility signatures identify potential antimicrobial targets in the Acinetobacter baumannii cell envelope
Source: Nat Commun. 2020 Sep 9;11:4522. doi: 10.1038/s41467-020-18301-2 (PMC7481262; doi:10.1038/s41467-020-18301-2)
Supplement: Supplementary file 1 — Supplementary Information [file 41467_2020_18301_MOESM1_ESM.pdf]

Supplementary Information file

Antibiotic susceptibility signatures identify potential antimicrobial targets in the *Acinetobacter baumannii* cell envelope

Geisinger et al.

Supplementary Figures 1-6

Supplementary Tables 1-5

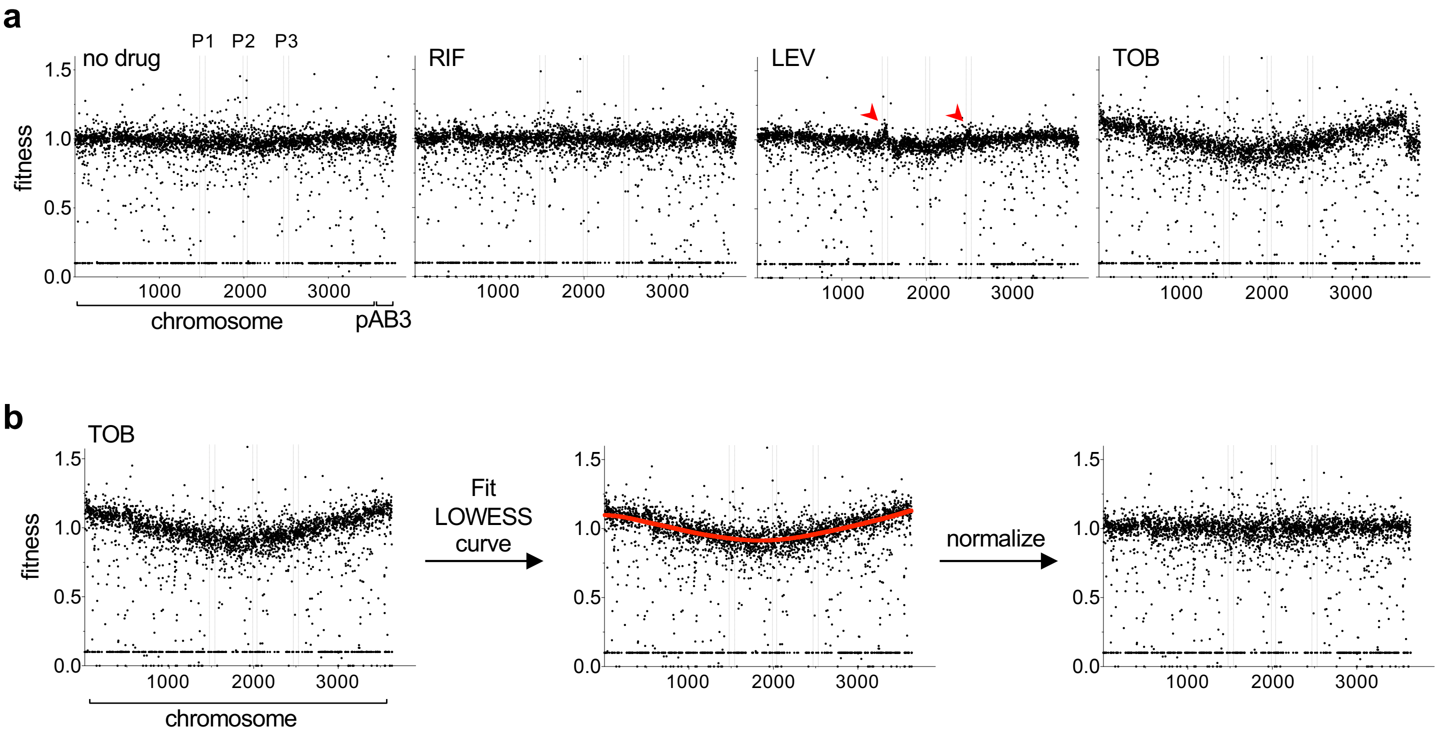

Supplementary Fig. 1. Correction of chromosome position bias in Tn-seq fitness values associated with TOB treatment. a, Average Tn-seq fitness scores for each gene were plotted according to gene order along the genome (chromosome and pAB3). Representative untreated (no drug) and treated (RIF) data are shown. Absence of large-scale chromosome position bias was confirmed with most treatments. Two exceptions were LEV and TOB. LEV resulted in slight local fitness value increases (red arrowheads) in the region of two prophages (P1 and P3, indicated by dotted lines). TOB caused fitness values to be higher for chromosomal genes closer to the origin of replication, and lower for those close to the replication terminus. **b**, To correct TOB-associated chromosome position bias, fitness values were normalized based on fitting to a LOWESS curve (red). Source data are provided as a Source Data file.

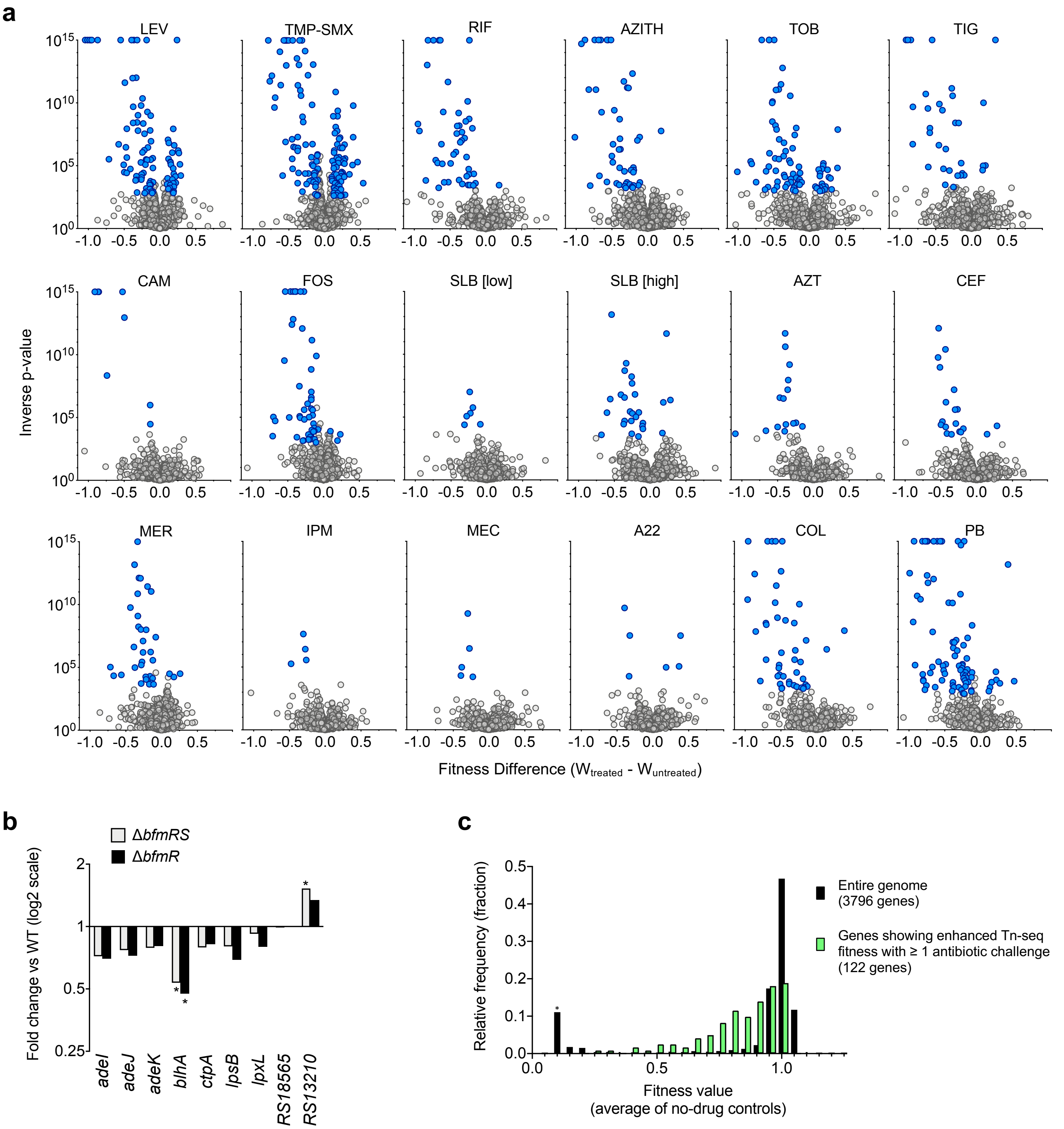

Supplementary Fig. 2. Intrinsic drug susceptibility determinants in *A. baumannii* identified through Tn-seq. a. Volcano plots show change in gene-level fitness between the indicated sub-MIC antibiotic treatment and untreated control plotted against inverse p value from parallel t tests. tested antibiotic. Blue data points indicate genes that represent candidate drug susceptibility determinants based on passing the three significance criteria (Materials and Methods). Grey data points indicate genes not passing significance criteria. **b.** BfmRS affects transcription of *blhA*. Using our published RNAseq data[1], candidate broad-susceptibility determining genes were examined for altered transcription levels in strains with *bfmRS* or *bfmR* deletion compared to WT control. Bars show fold change in RNAseq reads (mutant vs WT). *, $q < 0.05$. **c**, Large fraction of genes showing increased Tn-seq fitness with antibiotic challenge have fitness defect in absence of drug. Histogram shows fraction of genes (across entire genome, black; or within subset of 122 genes showing increased Tn-seq fitness with at least one antibiotic challenge, green) with the indicated fitness value in the absence of drug stress. Gene-level fitness values were averaged across all 12 untreated control conditions. Bin marked with * includes essential and/or small genes in which no transposon insertions were detected (assigned fitness of 0.1). Source data are provided as a Source Data file.

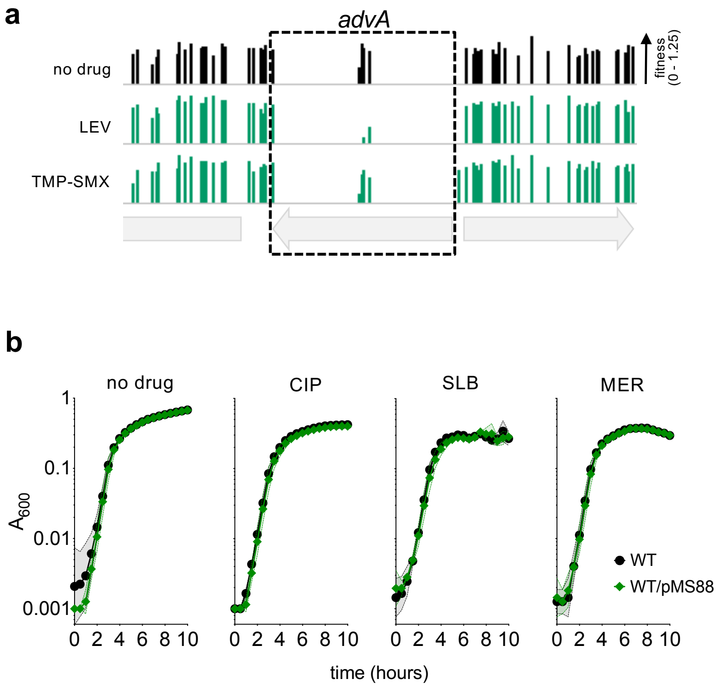

c

#	Template	Alignment Coverage	3D Model	Confidence	% i.d.	Template Information
1	c5o7jA_	Alignment	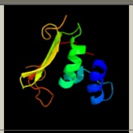	98.8	26	**PDB header:**signaling protein **Chain:** A: **PDB Molecule:**histidine kinase; **PDBTitle:** structural insights into the periplasmic sensor domain of the gacs2 histidine kinase controlling biofilm formation in pseudomonas3 aeruginosa
2	c4ywzA_	Alignment	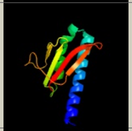	97.2	14	**PDB header:**transferase **Chain:** A: **PDB Molecule:**sensor protein kinase walk; **PDBTitle:** crystal structure of the extracellular receptor domain of the2 essential sensor kinase walk from staphylococcus aureus
3	d3by8a1	Alignment	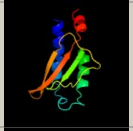	95.4	16	**Fold:**Profilin-like **Superfamily:**Sensory domain-like **Family:**Sensory domain of two-component sensor kinase
4	d1p0za_	Alignment	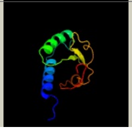	94.4	11	**Fold:**Profilin-like **Superfamily:**Sensory domain-like **Family:**Sensory domain of two-component sensor kinase

Supplementary Fig. 3. Additional information related to *advA* (ACX60_RS00475) mutant analysis. a, *Mariner* transposon insertions in *advA* yielding mutants with detectable fitness map to the same narrow region of the gene as observed with Tn*10* insertions. Bars show fitness values of individual *mariner* transposon mutants at the indicated locus across all tested banks as in Fig. 3. Transposon insertions in *advA* mapped to codons between residues 203 and 227. **b,** Presence of pMS88 does not affect growth with sub-MIC CIP, SLB, or MER. WT without vector, and WT harboring pMS88 vector were grown to early post-exponential phase at 30˚C, back-diluted into media with or without the indicated antibiotic at sub-MIC (Supplementary Table 1), and grown in microtiter format at 37˚C. Data points show geometric mean +/- s.d. (n = 3 independent cultures) as in Fig. 3b. Source data are provided as a Source Data file. **c,** Phyre2 homology modeling prediction of AdvA folding using ACX60_RS00475 protein sequence. Red horizontal lines in alignment coverage show where along the AdvA polypeptide (white) a structural homolog was found. Hits having >90% confidence that they result from true homology are shown.

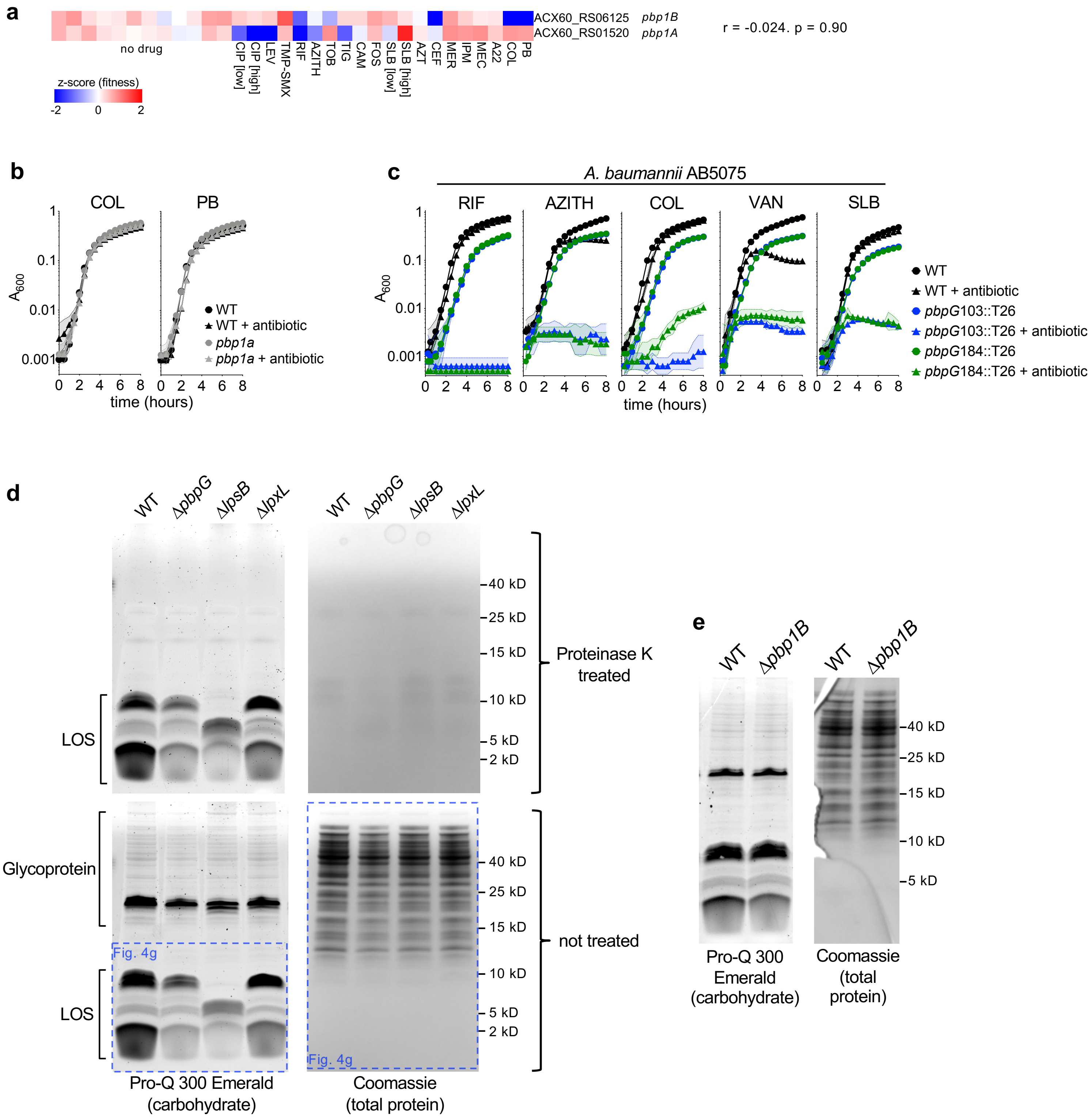

Supplementary Fig. 4. Additional analysis of relationship of hydrophobic/amphipathic compound sensitivity and LOS biosynthesis. a, *pbp1A* phenotypic signature does not correlate strongly with that of *pbp1B*. Heat map shows z-scored Tn-seq fitness values. Pearson r correlation and p-value of *pbp1A* and *pbp1B* phenotypic signatures are indicated. **b**, *pbp1A* mutation does not affect growth with COL or PB, as predicted by its Tn-seq phenotypic signature. EGA627 [*pbp1A*(N178TfsX27)] and WT control were grown in the absence or presence of the indicated antibiotic at 0.3 µg/ml and growth monitored in microtiter format. Data points show geometric mean +/- s.d. (n = 3 independent cultures) as in Fig. 3. **c**, *pbpG* inactivation lowers antibiotic susceptibility in a second *A. baumannii* strain, AB5075. Pure cultures of AB5075 WT, AB09564 (*pbpG*103::T26), and AB09566 (*pbpG*184::T26) were grown in the absence or presence of the indicated antibiotic at concentrations listing in Supplementary Table 1. Data points show geometric mean +/- s.d. (n = 3 independent cultures) as in Fig. 3. **d**, Proteinase-K treatment does not affect LOS banding pattern, allowing total protein to be used for normalization of LOS signals. Cell lysates were processed in parallel with (top) or without (bottom) proteinase-K treatment before separation via SDS-PAGE and staining for carbohydrate and total protein. Dotted blue rectangles indicate portions of the same gel shown in Fig. 4g. Approximate molecular weights (kDa) are indicated. **e**, WT and Δ*pbp1B* (EGA691) were analyzed for LOS content without proteinase-K treatment as described in d. Source data are provided as a Source Data file.

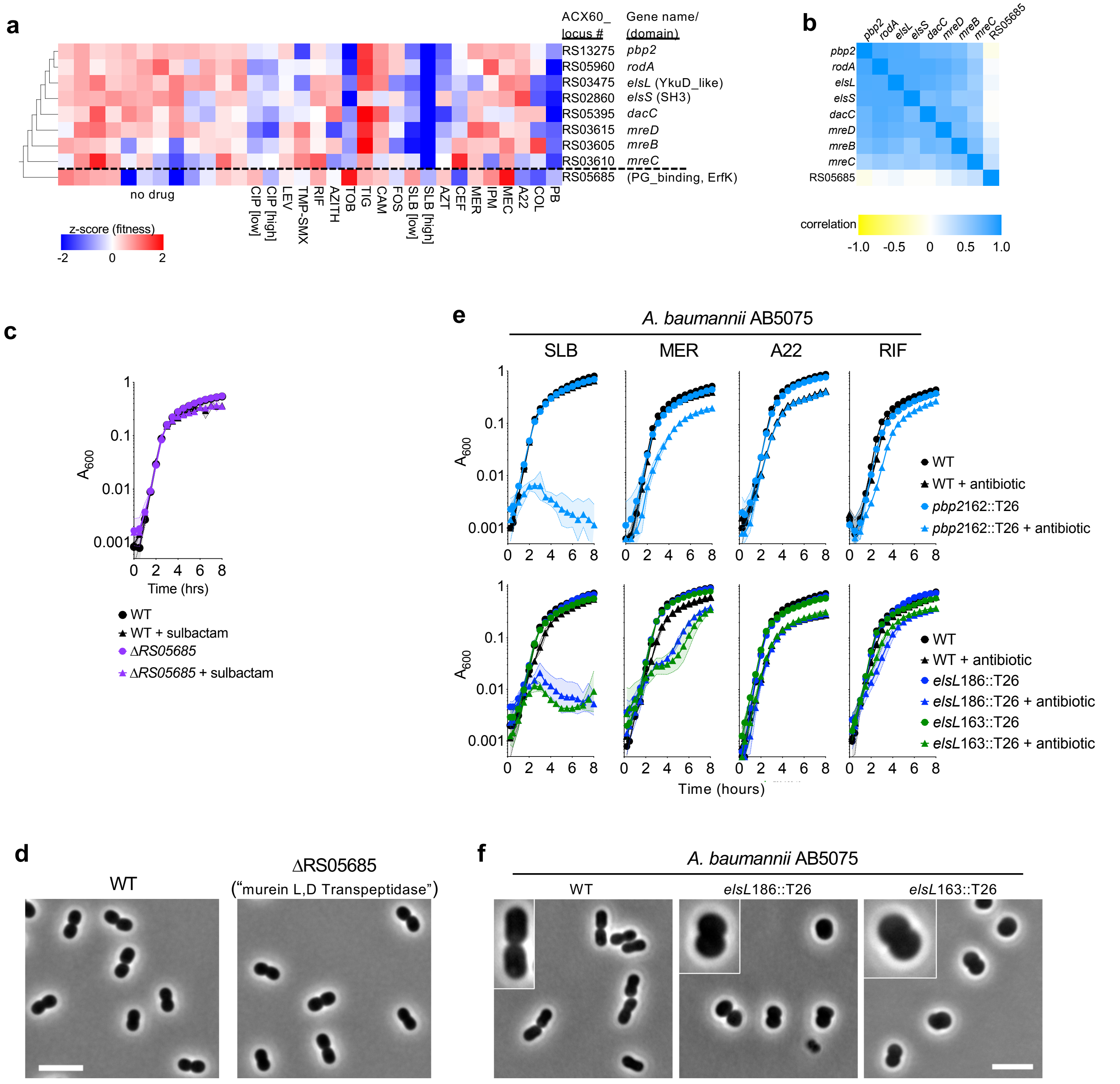

Supplementary Fig. 5. Additional analysis of relationship of susceptibility to specific block of cell wall synthesis machineries and cell shape. **a**, Tn-seq phenotypic signatures belonging to Rod system genes plus Fig. 5 discriminating genes identified based on preferential hypersusceptibility to cell wall inhibitors causing filamentation. Heat map shows Tn-seq fitness displayed as z-scored units. Dashed line separates the non-discriminating ACX60_RS05685 signature from those of other genes. **b**, Correlation matrix of Tn-seq fitness signatures. Heat map shows Pearson r values. **c**,**d**, ΔRS05685 mutant does not show SLB hypersensitivity or rod shape defect, consistent with the Tn-seq results. RS05685 refers to ACX60_ RS05685. Pure cultures of ATCC 17978 WT and EGA739 (ΔRS05685) were grown with or without SLB (0.3 µg/ml) as indicated and growth was monitored (c). Data points show geometric mean +/- s.d. (n = 3 independent cultures) as in Fig. 3. Mid-log phase cells without antibiotics were imaged via phase contrast microscopy (d). Scale bar, 5µm. **e**,**f**, Mutations in AB5075 strain background display phenotypes consistent with predictions from phenotypic signature analysis in ATCC 17978. AB5075 WT control and mutant strains AB07523, AB01891, and AB01892 (Supplementary Table 4) were cultured as above and data are shown as in panels c and d. Insets in f show 2x magnified views of representative bacteria. Scale bar, 5µm. Source data are provided as a Source Data file.

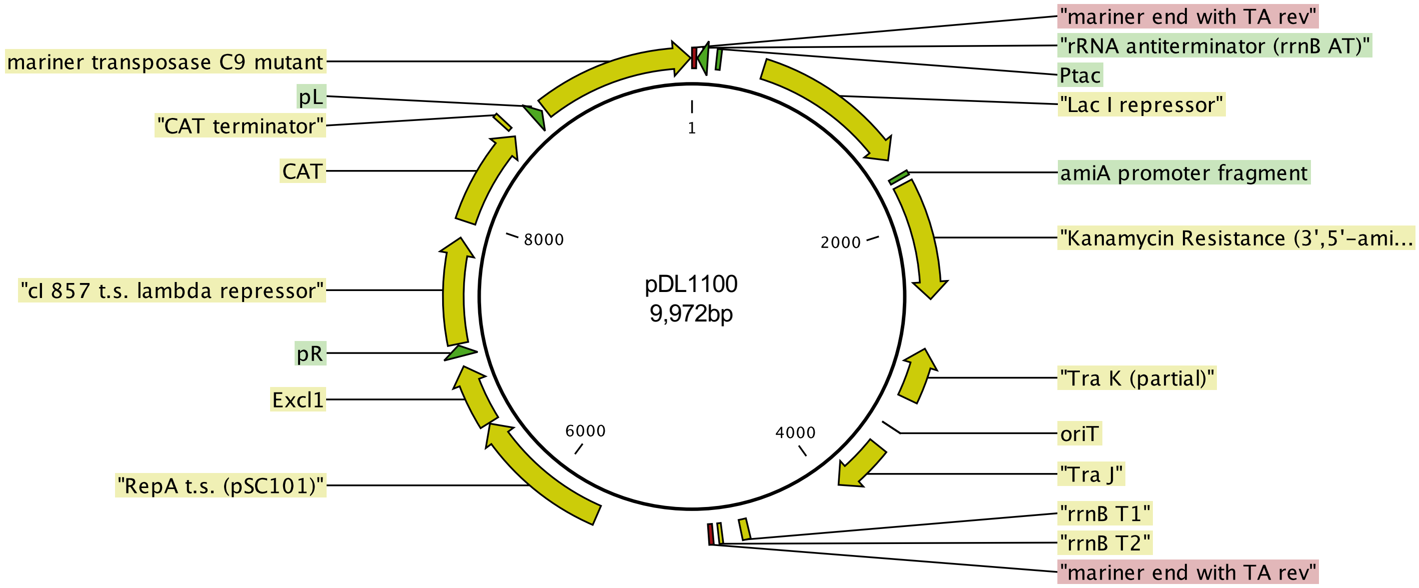

Supplementary Fig. 6. pDL1100 features map.

Supplementary Table 1. Information on antibiotics and transposon libraries used in these studies.

					Tn-seq (ATCC 17978)			Validation experiments	
	abbreviation	MW	xlogP3 (PubChem)	MIC in µg/ml (ATCC 17978)[a]	drug concentration (µg/ml)	growth rate relative to untreated[b]	transposon library used	drug concentration (µg/ml) with ATCC 17978	drug concentration (µg/ml) with AB5075
Colistin	COL	1155.5	-3.3	1	0.15-0.2	0.77	Tn*10*	0.3	0.3
Polymyxin B	PB	1203.5	-2.5	0.5	0.15-0.25	0.74	Tn*10*	0.3	ND
Rifampicin	RIF	822.9	4.9	4	0.65-0.85	0.79	Tn*10*	0.85	0.4
Azithromycin	AZITH	749.0	4	2	0.75-1	0.75	Tn*10*	0.2	2
Mecillinam	MEC	325.4	2.1	64	10-12	0.76	Tn*10*	16	ND
Aztreonam	AZT	435.4	0.3	16	6-8	0.76	Tn*10*	5	80
Sulbactam [high]	SLB	233.2	-1	1	0.25-0.3	0.72	Tn*10*	0.3	16
Sulbactam [low]	SLB	233.2	-1	1	0.15	0.86	Tn*10*		
Ceftazidime	CEF	546.6	0.4	4	2	0.76	Tn*10*	ND	64
Imipenem	IPM	299.3	-0.7	0.4	0.025	0.71	Tn*10*	0.035	ND
Meropenem	MER	383.5	-2.4	0.2	0.045-0.05	0.7	Tn*10*	0.05	4
A22	A22	271.6	ND	64	14-16	0.75	Tn*10*	16	10
Fosfomycin	FOS	138.1	-1.4	125	75-90	0.75	Tn*10*	ND	ND
Trimethoprim-Sulfamethoxazole[c]	TMP-SMX	290.3 / 253.3	0.9 / 0.9	16	8-9.5	0.74	*mariner*	ND	ND
Tobramycin	TOB	467.5	-6.2	2	0.75-1	0.78	Tn*10*	ND	ND
Tigecycline	TIG	585.7	1.1	0.5	0.15-0.25	0.73	Tn*10*	ND	ND
Chloramphenicol	CAM	323.1	1.1	128	8.5-9	0.74	Tn*10*	ND	ND
Ciprofloxacin [high][d]	CIP	331.3	-1.1	0.5	0.09-1	0.74	Tn*10*	ND	ND
Ciprofloxacin [low][d]	CIP	331.3	-1.1	0.5	0.075	0.84	Tn*10*		
Levofloxacin	LEV	361.4	-0.4	0.25	0.05-0.06	0.74	*mariner*	ND	ND
Vancomycin	VAN	1449.2	-3.1	500	ND	ND	ND	ND	100

[a]MIC determined in LB by CFE assay (1) except for AZITH, LEV, MER, TIG, and TMP/SMX (broth dilution)

[b]average of 10 independent cultures

[c]TMP-SMX denotes combination of Trimethoprim (TMP) and Sulfamethoxazole (SMX) in 1:5 ratio

[d]Tn-seq data with ciprofloxacin were described in (2)

ND, not determined

Supplementary Table 2. List of top-ranked antibiotic susceptibility determinants in which Tn mutation causes significantly decreased (blue) or increased (yellow) fitness with 10 or more antibiotic challenge conditions.

				Significant Tn-seq fitness change (drug treated vs untreated)																			
locus	Gene name	Protein ID	Protein annotation	CIP [low]	CIP [high]	LEV	TMP-SMX	RIF	AZITH	TOB	TIG	CAM	FOS	SLB [low]	SLB [high]	AZT	CEF	MER	IPM	MEC	A22	COL	PB
ACX60_RS03835	*adeI*	WP_000986589.1	Efflux system membrane fusion protein	blue	blue	blue	blue	blue			blue	blue				blue	blue	blue					
ACX60_RS03830	*adeJ*	WP_000046679.1	Efflux pump membrane transporter	blue	blue	blue	blue	blue	blue	blue	blue	blue			blue	blue	blue	blue					
ACX60_RS03825	*adeK*	WP_001174793.1	Efflux transporter, outer membrane factor lipoprotein	blue	blue	blue	blue	blue	blue	blue	blue	blue			blue	blue	blue	blue		blue		blue	blue
ACX60_RS14635	*bfmR*	WP_000076440.1	Two-component system response regulator protein	blue	blue	blue	blue	blue	blue	blue						blue		blue	blue	blue		blue	blue
ACX60_RS16945	*blhA*	WP_001207335.1	Uncharacterized protein	blue	blue	blue	blue			blue			blue	blue	blue	blue	blue	blue	blue	blue			blue
ACX60_RS16915	*ctpA*	WP_000939111.1	C-terminal processing peptidase family protein	blue	blue	blue	blue	blue	blue	yellow	blue		blue			blue	blue					blue	blue
ACX60_RS15945	*lpxL* $_{Ab}$	WP_000078875.1	Lipid A biosynthesis lauroyltransferase	blue	blue	blue	blue	blue	blue		blue					blue	blue					blue	blue
ACX60_RS15950	*lpsB*	WP_000867093.1	LOS glycosyl transferase		blue	blue		blue	blue						blue	blue	blue	blue				blue	blue
ACX60_RS13210		WP_001004359.1	5-formyltetrahydrofolate cyclo-ligase				blue			blue			blue	blue	blue	blue	blue	blue				blue	blue
ACX60_RS18565		WP_001101323.1	Uncharacterized protein (pAB3)		blue			blue		blue	blue		blue		blue		blue	blue	blue			blue	blue

blue = mutation of gene caused decreased fitness in presence of drug
yellow = mutation of gene caused increased fitness in presence of drug

Supplementary Table 3. Drug-drug interaction results.

	diagnoal method (duplicate determinations)			checkerboard (single determination)
drug combination	average log2FIC score	s.d.	p value (two tailed)[a]	alpha score
A22+AZT	-0.39	0.58	0.516	
A22+CEF	-0.44	0.30	0.292	
A22+COL	0.06	0.30	0.813	
A22+IPM	0.11	0.10	0.361	
A22+MEC	0.27	0.13	0.205	4.39
A22+SLB	-0.49	0.13	0.116	
**AZT+CEF**	**-0.85**	**0.06**	**0.034**	-0.19
AZT+COL	-0.96	0.40	0.181	
AZT+IPM	-0.20	0.03	0.064	
**AZT+MEC**	**-1.61**	**0.10**	**0.028**	-2.14
**AZT+SLB**	**-0.66**	**0.04**	**0.029**	-0.29
CEF+COL	0.32	0.10	0.137	
CEF+IPM	-0.39	0.33	0.339	
CEF+MEC	-0.69	0.35	0.216	-1.22
CEF+SLB	-0.06	0.08	0.500	0.06
COL+IPM	0.03	0.25	0.874	
COL+MEC	0.12	0.13	0.410	
COL+SLB	0.08	0.21	0.688	
IPM+MEC	0.06	0.09	0.500	-0.33
**IPM+SLB**	**-0.49**	**0.05**	**0.046**	
**MEC+SLB**	**-0.99**	**0.07**	**0.032**	-2.33

[a]Mean log2FIC score of each interaction was compared to a hypothetical mean of 0 via one-sample t-test. Scores significantly different from the reference value ($p < 0.05$) are highlighted in bold.

Supplementary Table 4. Strains and plasmids.

Strain or plasmid	Genotype or description	Reference
A. baumannii		
ATCC 17978	cerebrospinal fluid isolate	3
EGA746	ATCC 17978 Δ*blhA*	This study
EGA745	ATCC 17978 Δ*advA* with pEGE292 (pMS88::*advA*)	This study
AFA11	ATCC 17978 Δ*advA* with pEGE309 (T5*lac*P-*advA*-*gfp*)	This study
EGA749	ATCC 17978 Δ*pbpG*	This study
YDA341	ATCC 17978 Δ*pbpG* with pYDE210	This study
YDA265	ATCC 17978 Δ*lpsB*	This study
YDA269	ATCC 17978 Δ$lpxL_{Ab}$	This study
EGA691	ATCC 17978 Δ*pbp1B*	This study
EGA627	ATCC 17978 *pbp1A*(N178TfsX27)	2
EGA692	ATCC 17978 Δ*pbp2*	1
YDA208	ATCC 17978 Δ*pbp2* with pYDE135	1
EGA738	ATCC 17978 Δ*elsL* (ACX60_RS03475)	This study
EGA780	ATCC 17978 Δ*elsL* (ACX60_RS03475) with pEGE308	This study
EGA739	ATCC 17978 ΔACX60_RS05685	This study
YDA229	ATCC 17978 Δ*elsS* (ACX60_RS02860)	This study
AB5075-UW	bone isolate/osteomyelitis	4
AB09564	AB5075-UW *pbpG*103::T26 (Tn insertion at ABUW_3638 ORF position 737bp)	4
AB09566	AB5075-UW *pbpG*184::T26 (Tn insertion at ABUW_3638 ORF position 140bp)	4
AB07523	AB5075-UW *pbp2*162::T26 (Tn insertion at ABUW_2876 ORF position 443bp)	4
AB01891	AB5075-UW *elsL*186::T26 (Tn insertion at ABUW_0690 ORF position 239bp)	4
AB01892	AB5075-UW *elsL*163::T26 (Tn insertion at ABUW_0690 ORF position 333bp)	4
E. coli		
DH5α	*supE44* Δ*lacU169* (ϕ80lacZΔM15) *hsdR17 recA1 endA1 gyrA96 thi*-1 *relA1*	5
DH5λpir	DH5α (λpir) *tet*::Mu *recA*	6
TO60	DH5α λpir [F′ *proAB lacI*^{q}ZΔM15 Tn10 (Tc^R)]	7
XL1-blue	*recA1 endA1 gyrA96 thi-1 hsdR17 supE44 relA1 lac* [F′ *proAB lacI*^{q}ZΔM15 Tn10 Tc^R)]	Stratagene
BTH101	F−, *cya-99*, *araD139*, *galE15*, *galK16*, *rpsL1* (Sm^R), *hsdR2*, *mcrA1*, *mcrB1*	8
**plasmids**		
pUC18	*oriColE1* MCS Cb^R	9
pSR47S	*oriTRP4 oriR6K sacB* Km^R	10
pJB4648	Gm^R derivative of pSR47S	10
pMS88	Ts (stability) derivative of broad-host range plasmid R1162 (IncQ Su^R Sm^R Km^R)	11
pEGE292	pMS88 with *advA* replacing NheI-PstI fragment (Sm^R Km^R)	This study
pWH1266	*ori pBR322 ori pWH1277* MCS Tc^r Cb^r	12

pEGE305	Derivative of pWH1266 *E. coli*-*A. baumannii* shuttle vector with *lacI*^{q},T5-*lac*P, Tc^R	1
pEGE309	pEGE305 with *advA*-*gfp* downstream of T5-*lac*P promoter	This study
pEGE308	pEGE305 with *elsL* downstream of T5-*lac*P promoter	This study
pYDE135	pEGE305 with *pbp2* downstream of T5-*lac*P promoter	This study
pYDE153	pEGE305 with SacI-XbaI fragment removed and filled, and PstI-EcoRI fragment replaced with a linker containing multiple cloning sites, Tc^R	This study
pYDE210	pYDE153 with *pbpG* downstream of T5-*lac*P promoter	This study
pDL1073	Tn10 (Km^R) delivery plasmid, *ori pSC101* Cb^R	2
pDL1100	Mariner (Km^R) delivery plasmid, *ori pSC101* Cm^R	This study
pKT25	Bacterial two-hybrid plasmid for fusion of protein N-terminus with CyaA T25 fragment, *ori*p15, Km^R	8
pKNT25	Bacterial two-hybrid plasmid for fusion of protein C-terminus with CyaA T25 fragment, *ori*p15, Km^R	8
pUT18	Bacterial two-hybrid plasmid for fusion of protein C-terminus with CyaA T18 fragment, *oriColE1*, Cb^R	8
pUT18C	Bacterial two-hybrid plasmid for fusion of protein N-terminus with CyaA T18 fragment, *oriColE1*, Cb^R	8

Supplementary Table 5. Oligonucleotide primers

**Cloning**		
**primer name**	**Sequence (5’ – 3’; restriction site underlined)**	**RE site(s)**
gene deletion		
DadvA–upF	CAAGTGGATCCATCGCAAGCATCGGTAAATC	BamHI
DadvA–upR	GGCTAGCGGTACCCCCTTGTCTAGGCGCAATC	KpnI
DadvA–dwnF	CCTCAAGGTACCAGCCCGAGTAGTGCAGCATC	KpnI
DadvA–dwnR	CTTTATGTCGACTAAGCAAATCGCGAACAGGAC	SalI
DpbpGup–F	ACGGTAGGATCCATACCAAGTGCCCAAACG	BamHI
DpbpGup–R	AAAGCTGGTACCACTCAAACTAAGCAAAATAGACATGC	KpnI
DpbpGdwn–F	ACTAACGGTACCGGTTGGTTAAGTAATTTGCCAAAACG	KpnI
DpbpGdwn–R	GATGGGGTCGACGGCGCAAGCCATGATGTATG	SalI
D–lpsB–upF	TTCGTGGATCCGAAGTTTGTTTCCCTGAATGGTC	BamHI
D–lpsB–upR	GGGAGGGTACCCATCACTTTCATTGTTTTTTGCC	KpnI
D–lpsB–dwnF	CCCTTGGTACCTATCAAAGTGTATTGAATTGAAATAACCC	KpnI
D–lpsB–dwnR	ACAAAGCGGCCGCTCTCAATATAAGCATACAACTGAAGAAG	NotI
D–lpxL–upF	TTGAAGGGATCCTCCTTGAAAAGTTAGAGCGTTG	BamHI
D–lpxL–upR	GCGTGGTACCCTGCTTTTGGCTCATAACGATATAAAG	KpnI
D–lpxL–dwnF	CTGAGGGTACCGAAGAGATTTATTAAGATAAGGCTG	KpnI
D–lpxL–dwnR	CTTTGCGCGGCCGCGTGTTTTGCAACTCACTTAAATAG	NotI
D–elsL–dwnF	AGCAGTGGTACCGCCGCACAATTTCCTGAGCGAG	KpnI
D–elsL–dwnR	GCTCTAGTCGACGAGCTAAGCGCCCTTAACATCTTCATCG	SalI
D–elsL–upF	TACTCTGGATCCGAGACATAAATTGAAGTAAGGGTTCTG	BamHI
D–elsL–upR	TAAATTGGTACCTCGAATTTCTTCTCTTATATCAACTCAAC	KpnI
DldtAb2–dwnF	TTAGGTGGTACCGTACGTTCAGGCGTAACTGTTAAATTC	KpnI
DldtAb2–dwnR	ACAGTAGTCGACGCTTTAGATGCTTTTGAGAAAAAGAAGG	SalI
DldtAb2–upF	TTCACGGGATCCTAAAGCCCCACCCCAACTATC	BamHI
DldtAb2–upR	CATAGCGGTACCTGAGCGAACAAACATGTAATTCAAC	KpnI
D–elsS–upR	AGAAAGGTACCGGTTGCAAGCTGCATGCGTTTAC	KpnI
D–elsS–upF	TAAGACGCGGCCGCCAGCCGATACACAGCATCCTTTAC	NotI
D–elsS–dwnR	TGCTTGGATCCGCAATCCCAGCGCCAATTACG	BamHI
D–elsS–dwnF	CCAAAGGTACCCGTCAGTGGTAATCCTGAGCGAC	NotI
D–dacC–upF	TTCGTGGATCCTGCCATGTACCTAAGTGGTTATTC	BamHI
D–dacC–upR	GGGAGGGTACCTCGAGTCATTCTAGGTAATTCCAATATC	KpnI
D–dacC–dwnF	CTGAGGGTACCTTCAGCAACTTATTCTAAATTTAA	KpnI
D–dacC–dwnR	CTTTGCGCGGCCGCGCTTCTGTTAGTGTTTCACTACAT	NotI
gene expression *in trans*		
advA–1–F	TGAATTGACTCTAGTCTGAGTGACAAC	
advA–1–R	CGCTGTTTATAGAATTTTGCCTATG	
advA–bamF	CAACTAGGATCCGAATCATAATCGAATTTCAATATAATTGCCTTGG	BamHI
advA–xbatrlR	AATTTTCTAGATGCTGCACTACTCGGGCTGTC	XbaI
pbpG–F	AAATTAGAATTCCTATTCTTCTATAGTGAGCGAATAGTTG	EcoRI
pbpG–R	GTTGTCTCTAGATGCAACAATGGACCAAGTAAAAGATTCG	XbaI
pbp2–Feco	CATTATGAATTCCATTTTCCCTAATCGTATGGTG	EcoRI
pbp2–Rpst	ATAATCTGCAGTTGATTTTTCATTATTCATCGACCTC	PstI
elsL–F	GCAAGACAAGCTTTCCCATAATTTTTC	
elsL–R	GCACTTATTTTAATATTTTGATCATTCGGCT	
Bacterial Two–Hybrid		
elsS–2H–sphF	GGGTAAGCATGCACAGCTTGCAACCAAAAGCTTTCTTG	SphI
elsS–2H–xbaR	GTCGCTTCTAGAGACCACTGACGTCGACGTTTGGTGTAAAC	XbaI

mreD-2H-hindF	AGGAGAAGCTTGATGCCGATCGCTAAGTTGAAACG	HindIII
mreD-2H-xbaR	GAAGCTCTAGAGAATTGCGCCATTTTGCTAAACAATAATAGAC	XbaI
rodA-2H-pstF	AGTTACTGCAGCATGTCTCCTAGTCCACAATATAAATTTTTACGC	PstI
rodA-2H-xbaR	AAATTTCTAGAGATCGATGTGTATGAATAGACATGACTAAACCA	XbaI
pbp2-2H-xbaF	TAAGTCTCTAGAGATGAAACAGCACTTTCCTTTAAAAGATATCCAG	XbaI
pbp2-2H-ecoR	ATTGATGAATTCTTATTCATCGACCTCGTTTGTAGCAG	EcoRI
mreC-2H-xbaF	GTTTAATCTAGAGTCGGTGCAACCGAATATTTTTTCAAGACAGC	XbaI
mreC-2H-ecoR	TCTCAGAATTCAACTTAGCGATCGGCATATGGTTGC	EcoRI
pbp1bB2H-F-xba	TAGATCTAGAAATGAAGTTTGAACGTGGTATCG	XbaI
pbp1bB2H-R-eco	CTTTGAATTCTCCTGTTTCCTGTTAACGCT	EcoRI
pbp1aB2H-F-xba	TCTAGACATGAAAAAGCTATCCAGTTTGGG	XbaI
pbp1aB2H-R-eco	GAATTCGCGTAATAAAAAAGCCATCTAACGA	EcoRI
**Mariner Tn-seq sequencing library construction**		
**primer name**	**Sequence (5' – 3')**	**index**
First PCR		
Nextera 2A-R	GTCTCGTGGGCTCGGAGATGTGTATAAGAGACAG	
olj928	CTGTGTGGGCACTCGACATATGAC	
olj638	CTGTGTGGGCACTCGATGACGTCAGACC	
Second PCR -- Leftward TN10 specific Nextera Indexed primers		
Olk147	AATGATACGGCGACCACCGAGATCTACACGCAGGCGGCCGGGGGCCAAAATCATTAGGGGATTCATCAG	GCAGGCGG
Olk148	AATGATACGGCGACCACCGAGATCTACACAGGCAGAACCGGGGGCCAAAATCATTAGGGGATTCATCAG	AGGCAGAA
Olk149	AATGATACGGCGACCACCGAGATCTACACCAGAGAGGCCGGGGGCCAAAATCATTAGGGGATTCATCAG	CAGAGAGG
Olk150	AATGATACGGCGACCACCGAGATCTACACCGAGGCTGCCGGGGGCCAAAATCATTAGGGGATTCATCAG	CGAGGCTG
Olk151	AATGATACGGCGACCACCGAGATCTACACAAGAGGCACCGGGGGCCAAAATCATTAGGGGATTCATCAG	AAGAGGCA
Olk152	AATGATACGGCGACCACCGAGATCTACACGAGGAGCCCCGGGGGCCAAAATCATTAGGGGATTCATCAG	GAGGAGCC
Olk153	AATGATACGGCGACCACCGAGATCTACACAGCGCAGACCGGGGGCCAAAATCATTAGGGGATTCATCAG	AGCGCAGA
Olk110	AATGATACGGCGACCACCGAGATCTACACGTAAGGAGCCGGGGGCCAAAATCATTAGGGGATTCATCAG	GTAAGGAG
Olk154	AATGATACGGCGACCACCGAGATCTACACAACCCGGACCGGGGGCCAAAATCATTAGGGGATTCATCAG	AACCCGGA
Olk155	AATGATACGGCGACCACCGAGATCTACACGCGGAAGCCCGGGGGCCAAAATCATTAGGGGATTCATCAG	GCGGAAGC
Second PCR -- Leftward Mariner specific Nextera Indexed primers		
mar147	AATGATACGGCGACCACCGAGATCTACACGCAGGCGGCGTTGACCGGGGACTTATCAGCCAACCTGTTA	GCAGGCGG
mar148	AATGATACGGCGACCACCGAGATCTACACAGGCAGAACGTTGACCGGGGACTTATCAGCCAACCTGTTA	AGGCAGAA
mar149	AATGATACGGCGACCACCGAGATCTACACCAGAGAGGCGTTGACCGGGGACTTATCAGCCAACCTGTTA	CAGAGAGG
mar150	AATGATACGGCGACCACCGAGATCTACACCGAGGCTGCGTTGACCGGGGACTTATCAGCCAACCTGTTA	CGAGGCTG
mar151	AATGATACGGCGACCACCGAGATCTACACAAGAGGCACGTTGACCGGGGACTTATCAGCCAACCTGTTA	AAGAGGCA
mar152	AATGATACGGCGACCACCGAGATCTACACGAGGAGCCCGTTGACCGGGGACTTATCAGCCAACCTGTTA	GAGGAGCC
mar153	AATGATACGGCGACCACCGAGATCTACACAGCGCAGACGTTGACCGGGGACTTATCAGCCAACCTGTTA	AGCGCAGA
mar110	AATGATACGGCGACCACCGAGATCTACACGTAAGGAGCGTTGACCGGGGACTTATCAGCCAACCTGTTA	GTAAGGAG
mar154	AATGATACGGCGACCACCGAGATCTACACAACCCGGACGTTGACCGGGGACTTATCAGCCAACCTGTTA	AACCCGGA
mar155	AATGATACGGCGACCACCGAGATCTACACGCGGAAGCCGTTGACCGGGGACTTATCAGCCAACCTGTTA	GCGGAAGC
Second PCR -- Rightward Nextera Indexed primers		
olk141	CAAGCAGAAGACGGCATACGAGATCCGCCTGCGTCTCGTGGGCTCGGAGATGTG	GCAGGCGG
N703 index	CAAGCAGAAGACGGCATACGAGATTTCTGCCTGTCTCGTGGGCTCGGAGATGTG	AGGCAGAA
N708 index	CAAGCAGAAGACGGCATACGAGATCCTCTCTGGTCTCGTGGGCTCGGAGATGTG	CAGAGAGG
N710 index	CAAGCAGAAGACGGCATACGAGATCAGCCTCGGTCTCGTGGGCTCGGAGATGTG	CGAGGCTG
N711 index	CAAGCAGAAGACGGCATACGAGATTGCCTCTTGTCTCGTGGGCTCGGAGATGTG	AAGAGGCA
olk142	CAAGCAGAAGACGGCATACGAGATGGCTCCTCGTCTCGTGGGCTCGGAGATGTG	GAGGAGCC
olk143	CAAGCAGAAGACGGCATACGAGATTCTGCGCTGTCTCGTGGGCTCGGAGATGTG	AGCGCAGA
olk144	CAAGCAGAAGACGGCATACGAGATCTCCTTACGTCTCGTGGGCTCGGAGATGTG	GTAAGGAG
olk145	CAAGCAGAAGACGGCATACGAGATTCCGGGTTGTCTCGTGGGCTCGGAGATGTG	AACCCGGA

olk146	CAAGCAGAAGACGGCATACGAGATGCTTCCGCGTCTCGTGGGCTCGGAGATGTG	GCGGAAGC
Reconditioning		
P1	AATGATACGGCGACCACCGA	
P2	CAAGCAGAAGACGGCATACGA	
Sequencing		
olk115	CCGGGGGCCAAAATCATTAGGGGATTCATCAG	
mar512	CGTTGACCGGGGACTTATCAGCCAACCTGTTA	

Supplementary References

1. Geisinger, E., Mortman, N. J., Vargas-Cuebas, G., Tai, A. K. & Isberg, R. R. A global regulatory system links virulence and antibiotic resistance to envelope homeostasis in *Acinetobacter baumannii*. *PLoS Pathog* **14**, e1007030, doi:10.1371/journal.ppat.1007030 (2018).
2. Geisinger, E. *et al.* The Landscape of Phenotypic and Transcriptional Responses to Ciprofloxacin in *Acinetobacter baumannii*: Acquired Resistance Alleles Modulate Drug-Induced SOS Response and Prophage Replication. *MBio* **10**, doi:10.1128/mBio.01127-19 (2019).
3. Bouvet, P. & Grimont, P. Taxonomy of the Genus Acinetobacter with the Recognition of *Acinetobacter baumannii* sp. nov. *Acinetobacter haemolyticus* sp. nov. *Acinetobacter johnsonii* sp. nov. and *Acinetobacter junii* sp. nov. and Emended Descriptions of *Acinetobacter calcoaceticus* and *Acinetobacter lwofii*. *International Journal of Systematic Bacteriology* **36**, 228-240 (1986).
4. Gallagher, L. A. *et al.* Resources for Genetic and Genomic Analysis of Emerging Pathogen *Acinetobacter baumannii*. *J Bacteriol* **197**, 2027-2035, doi:10.1128/JB.00131-15 (2015).
5. Hanahan, D., Jessee, J. & Bloom, F. R. Plasmid transformation of *Escherichia coli* and other bacteria. *Methods Enzymol* **204**, 63-113, doi:0076-6879(91)04006-A [pii] (1991).
6. Kolter, R., Inuzuka, M. & Helinski, D. R. Trans-complementation-dependent replication of a low molecular weight origin fragment from plasmid R6K. *Cell* **15**, 1199-1208, doi:0092-8674(78)90046-6 [pii] (1978).
7. O'Connor, T. J., Adepoju, Y., Boyd, D. & Isberg, R. R. Minimization of the *Legionella pneumophila* genome reveals chromosomal regions involved in host range expansion. *Proc Natl Acad Sci U S A* **108**, 14733-14740 (2011).
8. Karimova, G., Gauliard, E., Davi, M., Ouellette, S. P. & Ladant, D. Protein-Protein Interaction: Bacterial Two-Hybrid. *Methods Mol Biol* **1615**, 159-176, doi:10.1007/978-1-4939-7033-9_13 (2017).
9. Yanisch-Perron, C., Vieira, J. & Messing, J. Improved M13 phage cloning vectors and host strains: nucleotide sequences of the M13mp18 and pUC19 vectors. *Gene* **33**, 103-119 (1985).
10. Andrews, H. L., Vogel, J. P. & Isberg, R. R. Identification of linked *Legionella pneumophila* genes essential for intracellular growth and evasion of the endocytic pathway. *Infect Immun* **66**, 950-958 (1998).
11. Meyer, R., Hinds, M. & Brasch, M. Properties of R1162, a broad-host-range, high-copy-number plasmid. *J Bacteriol* **150**, 552-562 (1982).
12. Hunger, M., Schmucker, R., Kishan, V. & Hillen, W. Analysis and nucleotide sequence of an origin of DNA replication in *Acinetobacter calcoaceticus* and its use for *Escherichia coli* shuttle plasmids. *Gene* **87**, 45-51 (1990).
