# Supplementary material for: Antibiotic susceptibility signatures identify potential antimicrobial targets in the Acinetobacter baumannii cell envelope
Source: Nat Commun. 2020 Sep 9;11:4522. doi: 10.1038/s41467-020-18301-2 (PMC7481262; doi:10.1038/s41467-020-18301-2)
Supplement: Supplementary file 2 — Description of Additional Supplementary Files [file 41467_2020_18301_MOESM2_ESM.pdf]

**Description of Additional Supplementary Files**

File Name: Supplementary Data 1
Description: Genome-wide *A. baumannii* Tn-seq fitness values across 20 antibiotic treatments and 12 untreated control conditions. Spreadsheet shows matrix of Tn-seq fitness data across all conditions for loci encoded in *A. baumannii* strain 17978 chromosome and pAB3. Average fitness (W), s.d., and n (number of transposon mutant fitness values used to calculate W) are listed for each gene per condition. Genes not represented by transposon mutants were assigned W = 0.1. Gene annotations are listed in the first 5 columns and were obtained by querying NCBI Protein ID with KEGG Automatic Annotation server (gene name and protein annotation) and with UniProt (additional protein annotation). Condition information, including sample information and annotations used in discriminating variable analyses, are listed in rows 2-10. The "sample name" annotation distinguishes the different untreated control samples according to the corresponding treatment(s) that were performed in parallel. CIP data were described previously[2]. Untx, untreated control. NA, not applicable.

File Name: Supplementary Data 2
Description: Candidate antibiotic susceptibility determinants identified by Tn-seq fitness differences. (A) Data used to identify candidate determinants of susceptibility to 20 antibiotic conditions are listed. Fitness (Mean) in treated and untreated (untx) conditions; difference in mean fitness (treated-untreated, $W_{diff}$); and statistical analysis of fitness comparisons are listed for each antibiotic treatment experiment. Genes with no fitness value entered in untreated conditions had no transposon mutants represented across the mutant pools. (B) Candidate antibiotic susceptibility determinants. Genes are shown in which the corresponding transposon mutants shows significantly decreased or increased fitness in at least one drug treatment condition compared to no drug control based on significance criteria (Materials and Methods). Candidates are listed in rank order based on number of antibiotic conditions in which the mutant showed a fitness change. (C) Candidate antibiotic susceptibility determinants in which transposon mutation causes significantly decreased fitness in at least one antibiotic condition. Significance criteria were the same as in B, but with $W_{diff} < -0.1$. (D) Candidate antibiotic susceptibility determinants in which transposon mutation causes significantly increased fitness. Significance criteria were the same as in B, but with $W_{diff} > 0.1$.

File Name: Supplementary Data 3
Description: Functional information on ATCC 17978 genes obtained from querying KEGG and UniProt databases. Source of information in each column are as follows: Gene and Protein ID are from Genbank. K Number, Global Gene (KEGG Name), Global Product (KEGG Definition), Tags (KEGG Level 1 name), Categories (KEGG Level 2 Name), and Global Path (any subpathways involved) are from KEGG database. Tags and Categories are recorded as "Various" if greater than 3 were listed for a given gene. Entry (UniProt identifier), query (query used which is the same as the NCBI protein ID), Entry name (species specific extended UniProt Identifier), Protein names, Gene names, Organism, Length, EC number, and GO terms are from UniProt database.
