# Supplementary material for: Antibiotic susceptibility signatures identify potential antimicrobial targets in the Acinetobacter baumannii cell envelope
Source: Nat Commun. 2020 Sep 9;11:4522. doi: 10.1038/s41467-020-18301-2 (PMC7481262; doi:10.1038/s41467-020-18301-2)
Supplement: Supplementary file 6 — Reporting Summary [file 41467_2020_18301_MOESM6_ESM.pdf]

nature research

Corresponding author(s): Edward Geisinger, Ralph Isberg

Last updated by author(s): Aug 6, 2020

Reporting Summary

Nature Research wishes to improve the reproducibility of the work that we publish. This form provides structure for consistency and transparency in reporting. For further information on Nature Research policies, see our Editorial Policies and the Editorial Policy Checklist.

Statistics

For all statistical analyses, confirm that the following items are present in the figure legend, table legend, main text, or Methods section.

n/a	Confirmed	
☐	☒	The exact sample size (*n*) for each experimental group/condition, given as a discrete number and unit of measurement
☐	☒	A statement on whether measurements were taken from distinct samples or whether the same sample was measured repeatedly
☐	☒	The statistical test(s) used AND whether they are one- or two-sided<br>*Only common tests should be described solely by name; describe more complex techniques in the Methods section.*
☒	☐	A description of all covariates tested
☐	☒	A description of any assumptions or corrections, such as tests of normality and adjustment for multiple comparisons
☐	☒	A full description of the statistical parameters including central tendency (e.g. means) or other basic estimates (e.g. regression coefficient) AND variation (e.g. standard deviation) or associated estimates of uncertainty (e.g. confidence intervals)
☐	☒	For null hypothesis testing, the test statistic (e.g. *F*, *t*, *r*) with confidence intervals, effect sizes, degrees of freedom and *P* value noted<br>*Give P values as exact values whenever suitable.*
☒	☐	For Bayesian analysis, information on the choice of priors and Markov chain Monte Carlo settings
☒	☐	For hierarchical and complex designs, identification of the appropriate level for tests and full reporting of outcomes
☐	☒	Estimates of effect sizes (e.g. Cohen's *d*, Pearson's *r*), indicating how they were calculated

Our web collection on statistics for biologists contains articles on many of the points above.

Software and code

Policy information about availability of computer code

Data collection	Illumina Experiment Manager for sequencing read data; i-control (Tecan) and Gen-5 (2, Biotek) for microplate reader data; LAS X (Leica) and Openlab (Perkin Elmer) for microscopy data; and Image Lab (6.0.1, Bio Rad) for gel imaging data.
Data analysis	BBDuk, FASTX-toolkit, and Bowtie (1.1.2) for sequencing read mapping; custom scripts for fitness calculations (available at https://github.com/jsa-aerial/aerobio and https://github.com/vanOpijnenLab/MAGenTA); Qlucore Omics Explorer (3.5) for PCA and hierarchical cluster analysis, Cluster (3.0) for hierarchical cluster analysis; Integrative Genomics Viewer (2.4.5) for visualizing mutant fitness vs transposon insertion genomic coordinate; Magellan (7) and Gen-5 (2) for microplate reader data; Image Lab (6.0.1, Bio Rad) for gel imaging data; MATLAB (Mathworks) for reconstituting and analyzing drug-drug interaction plate measurement data; Prism (8) for statistical and graphical analyses; BRESEQ (0.28.1) for analysis of whole-genome re-sequencing data; SignalP (5.0), Phobius (1.01), CCTOP (s.1.00), Phyre (2.0) for protein domain/fold prediction.

For manuscripts utilizing custom algorithms or software that are central to the research but not yet described in published literature, software must be made available to editors and reviewers. We strongly encourage code deposition in a community repository (e.g. GitHub). See the Nature Research guidelines for submitting code & software for further information.

Data

Policy information about availability of data

All manuscripts must include a data availability statement. This statement should provide the following information, where applicable:

- Accession codes, unique identifiers, or web links for publicly available datasets
- A list of figures that have associated raw data
- A description of any restrictions on data availability

Source data are provided with this paper. All sequence data can be found in the NCBI Sequence Read Archive under accession codes: PRJNA485788 [https://

www.ncbi.nlm.nih.gov/bioproject/PRJNA485788], PRJNA485840 [https://www.ncbi.nlm.nih.gov/bioproject/PRJNA485840], PRJNA486258 [https://www.ncbi.nlm.nih.gov/bioproject/PRJNA486258], PRJNA486803 [https://www.ncbi.nlm.nih.gov/bioproject/PRJNA486803], PRJNA488082 [https://www.ncbi.nlm.nih.gov/bioproject/PRJNA488082], PRJNA636211 [https://www.ncbi.nlm.nih.gov/bioproject/PRJNA636211], PRJNA637031 [https://www.ncbi.nlm.nih.gov/bioproject/PRJNA637031], PRJNA638415 [https://www.ncbi.nlm.nih.gov/bioproject/PRJNA638415], PRJNA638316 [https://www.ncbi.nlm.nih.gov/bioproject/PRJNA638316], PRJNA639060 [https://www.ncbi.nlm.nih.gov/bioproject/PRJNA639060], PRJNA638887 [https://www.ncbi.nlm.nih.gov/bioproject/PRJNA638887], PRJNA485590 [https://www.ncbi.nlm.nih.gov/bioproject/PRJNA485590]. Publicly available protein functional information databases used were UniProt [https://www.uniprot.org/] and KEGG [https://www.genome.jp/kegg/]. Additional datasets are present in the article and Supplementary Information files. All other data and all genetic material used for this paper are available from the authors on reasonable request.

Field-specific reporting

Please select the one below that is the best fit for your research. If you are not sure, read the appropriate sections before making your selection.

☒ Life sciences ☐ Behavioural & social sciences ☐ Ecological, evolutionary & environmental sciences

For a reference copy of the document with all sections, see nature.com/documents/nr-reporting-summary-flat.pdf

Life sciences study design

All studies must disclose on these points even when the disclosure is negative.

Sample size	Sample size, which represents the number of biologically independent replicate samples in our study, was chosen to allow a sufficient number to make statistical inferences, and was also based on practical feasibility of the experiment.
Data exclusions	No data were excluded.
Replication	Findings from experiments with defined mutants were successfully reproduced in at least 2 replicate experiments on separate days. Tn-seq experiments used multiple independent transposon libraries challenged on separate days. Drug-drug interaction experiments using the diagonal method were performed twice, and findings were confirmed with a separate assay (checkerboard). Experiments with defined mutants used at least 3 independent cultures (biological replicates), except where noted in legend to Fig. 4c. Tn-seq experiments used at least 10 separate transposon pools cultured and processed independently to increase the number of mutants analyzed per gene, enhance reliability of fitness measurements, and minimize experimental noise.
Randomization	Allocation of samples into experimental groups was random. In drug-drug interaction experiments, drugs were printed using randomized dispense locations to minimize plate position effects.
Blinding	Blinding was not deemed applicable in our study because measurements were objective and quantitative.

Reporting for specific materials, systems and methods

We require information from authors about some types of materials, experimental systems and methods used in many studies. Here, indicate whether each material, system or method listed is relevant to your study. If you are not sure if a list item applies to your research, read the appropriate section before selecting a response.

Materials & experimental systems

n/a	Involved in the study	
☒	☐	Antibodies
☒	☐	Eukaryotic cell lines
☒	☐	Palaeontology and archaeology
☒	☐	Animals and other organisms
☒	☐	Human research participants
☒	☐	Clinical data
☒	☐	Dual use research of concern

Methods

n/a	Involved in the study	
☒	☐	ChIP-seq
☒	☐	Flow cytometry
☒	☐	MRI-based neuroimaging
